# Osmotolerant plant growth promoting bacteria mitigate adverse effects of drought stress on wheat growth

**DOI:** 10.3934/microbiol.2024025

**Published:** 2024-07-09

**Authors:** Naoual Bouremani, Hafsa Cherif-Silini, Allaoua Silini, Nour El Houda Rabhi, Ali Chenari Bouket, Lassaad Belbahri

**Affiliations:** 1 Laboratory of Applied Microbiology, Department of Microbiology, Faculty of Natural and Life Sciences, University Ferhat Abbas of Setif-1, 19000 Setif, Algeria; 2 Department of Natural Sciences and Life, Abdelhafid Boussouf University Center-Mila, 43000 Mila, Algeria; 3 East Azarbaijan Agricultural and Natural Resources Research and Education Centre, Plant Protection Research Department, Agricultural Research, Education and Extension Organization (AREEO), Tabriz 5355179854, Iran; 4 University Institute of Teacher Education (IUFE), University of Geneva, 24 Rue du Général-Dufour, 1211 Geneva, Switzerland

**Keywords:** drought tolerance, PGPB, durum wheat, water stress, abiotic stress

## Abstract

Drought stress represents a major constraint with significant impacts on wheat crop globally. The use of plant growth-promoting bacteria (PGPB) has emerged as a promising strategy to alleviate the detrimental impacts of water stress and enhance plant development. We investigated 24 strains from diverse ecosystems, assessed for PGP traits and tolerance ability to abiotic stresses (drought, salinity, temperature, pH, heavy metals, pollutants, herbicides, and fungicides). The most effective bacterial strains *Providencia vermicola* ME1, *Pantoea agglomerans* Pa, *Pseudomonas knackmussi* MR6, and *Bacillus* sp D13 were chosen. Furthermore, these strains exhibited PGP activities under osmotic stress (0, 10, 20, and 30% PEG-6000). The impact of these osmotolerant PGPBs on wheat (*Triticum durum* L.) growth under drought stress was assessed at two plant growth stages. In an *in vitro* wheat seed germination experiment, bacterial inoculation significantly enhanced germination parameters. In pot experiments, the potential of these bacteria was evaluated in wheat plants under three treatments: Well-watered (100% field capacity), moderate stress (50% FC), and severe stress (25% FC). Results showed a significant decline in wheat growth parameters under increasing water stress for uninoculated seedlings. In contrast, bacterial inoculation mitigated these adverse effects, significantly improving morphological parameters and chlorophyll pigment contents under the stress conditions. While malondialdehyde (lipid peroxidation) and proline contents increased significantly with drought intensity, they decreased after bacterial inoculation. The antioxidant enzyme activities (GPX, CAT, and SOD) in plants decreased after bacterial inoculation. The increased root colonization capacity observed under water stress was attributed to their ability to favorable adaptations in a stressful environment. This study highlighted the potential of selected PGPB to alleviate water stress effects on wheat, promoting practical applications aimed at enhancing crop resilience under conditions of water shortage.

## 1. Introduction

Agriculture is a critically important economic sector. Promoting its development is one of the most potent means to eradicate extreme poverty, foster shared prosperity, and sustain the needs of a rapidly expanding global population. Additionally, agriculture plays a vital role in the economic growth of certain developing countries, significantly influencing the income of many individuals worldwide.

The demand for food is on a constant rise due to the alarming rate of global population growth. According to forecasts from the Food and Agriculture Organization of the United Nations (FAO), the world population is expected to reach 8.6 billion in 2030, 9.8 billion in 2050, and 11.2 billion in 2100 [1]–[3]. To meet this escalating demand, agricultural production must increase by 60% to 70% by 2050 [2],[4]. However, this ambitious goal faces a significant obstacle-climate change.

Climate change poses a serious threat to global food security by adversely affecting plant production, particularly through increased abiotic stresses [5]. The rise in temperatures, altered precipitation patterns and more frequent extreme events contribute to worsening food insecurity [2],[6]–[8].

Wheat occupies a preeminent position among the cereals essential to human nutrition globally. As the third most significant food crop, wheat plays a crucial role in global food security. It covers 15% of the world's cultivated land (200,000,000 ha), surpassing all other plants in terms of surface area. This versatile plant adapts to a wide variety of climatic conditions, allowing it to grow in humid, subhumid, semi-arid and arid environments [4],[9]. Climate, with all its variables, is identified as the main factor influencing wheat productivity. Among these variables, water in the form of rain occupies a preponderant place, followed closely by temperature.

Drought stress, a multidimensional challenge, induces physiological, morphological, biochemical, and molecular changes in plants. Sporadic episodes of drought stress during critical growth stages can lead to a substantial reduction in yields and, in some cases, even harvest failure [8],[9].

Seed germination, a sensitive stage in a plant's life cycle, is particularly affected by water stress, leading to low germination rates and poor crop establishment [10]. Drought stress manifests in plants through reduced water pressure, stomatal closure, and the generation of reactive oxygen species, negatively impacting photosynthesis, metabolic processes, and cell growth [4],[6],[8],[11].

Addressing these challenges is decisive for global food security. While various solutions exist, such as developing drought-tolerant crop varieties and improving irrigation efficiency, many are expensive and time-consuming [1]. Plant growth-promoting bacteria (PGPB) offer a promising, cost-effective, and environmentally friendly alternative [4]. Studies showed that inoculating crops with PGPB resistant to osmotic stress and possessing plant growth-promoting activities can alleviate stress effects and enhance agricultural productivity [12]. This approach has been successful in improving growth and drought tolerance in various crops, including wheat (*Triticum aestivum* L.) [9],[12],[13], corn (*Zea mays* L.) [8], rice (*Oryza sativa* L.) [14],[15], barley (*Hordeum vulgare* L.) [16], sorghum (*Sorghum bicolor* L.) [17], and soybean (*Glycine max* L.) [18].

Plant Growth-Promoting Bacteria (PGPB) employ diverse mechanisms to enhance plant drought resistance directly and indirectly. They stimulate root growth and modify their architecture, promoting increased water and nutrient absorption, including nitrogen, zinc, iron, phosphorus, and potassium [3],[12]. The PGPB influence plant physiology by inducing the synthesis of hormones, activating signaling pathways, regulating antioxidant defense, and modulating stress response genes [19]. By counteracting reactive oxygen species and reducing ethylene levels through ACC deaminase [20], PGPB foster root surface expansion and overall plant development under stress conditions [3]. Additionally, PGPB's ability to form biofilms, characterized by extracellular polysaccharides (EPS), enhances bacterial drought tolerance and contributes to plant resilience. Biofilm formation improves water availability in the root zone, increasing cell viability and facilitating bacterial colonization of roots [21]. PGPB further induces systemic tolerance by producing metabolites and small molecules, including volatile organic compounds, thereby reinforcing the plant's ability to withstand drought [3],[22].

Therefore, searching for the most drought-tolerant PGPB is very beneficial for creating new inoculants in arid and semi-arid regions. In this study, several bacterial isolates, from different collections and multiple sites, were evaluated for their promoting plant growth traits and tolerance to abiotic stresses. The selected bacteria were tested for their ability to maintain promoting plant growth abilities under osmotic stress. Therefore, they were used as bioinoculants to improve the germination and growth of durum wheat (*Triticum durum* L.) under water-stress conditions.

## 2. Materials and methods

### 2.1. Biological material

The bacterial strains (n = 24) used in this study came from several collections from the Applied Microbiology Laboratory (Ferhat Abbas University, Sétif, Algeria). They were isolated from several ecosystems (Table 1) and were chosen based on their multiple performances.

**Table 1.** Bacterial isolates, strain names, and sampling sites.

Bacterial isolates	Sampling sites	References
B6, B10, B16, B22, B25, B28, D12, D13	Rhizospheric soil of Wheat and barley	[23]
BR5, OR15, RB13	Rhizosphere of halophytes from salt-affected soils	[24]
ET11, RK2	Rhizosphere of Terfezia	[25]
S260, GF17	Rhizosphere of date palm	[26]
Pa	Rhizospheric soil of Wheat	[27]
ME1, MN6, MN8, MR1, MR4, MR6, MR8	Rhizosphere and endophytes of halophytes from salt-affected soils	[28]
*Bacillus amyloliquefaciens* FZB42	Type strain	[29]

### 2.2. Measurement of PGP activities and abiotic stress tolerance

#### 2.2.1. Phosphate solubilization

A quantitative analysis of phosphate solubilization was carried out in a liquid medium according to Olsen et al. [30]. Briefly, absorbance was measured at 610 nm and compared to a standard calibration curve using KH_2_PO_4_ solution.

#### 2.2.2. Siderophore production

Siderophore production was screened according to Schwyn and Neilands [31], using Chrome Azurol S (CAS) medium. Siderophore production (SP) was evaluated using the Eq 1 described in Cherif-Silini et al. [27] and expressed as a percentage:

SP (%)=OD of sample/OD of control (CAS solution) (1)

#### 2.2.3. Indole Acetic Acid (IAA) production

Winogradsky salt (WS) medium containing 2 g/L of tryptophan was inoculated and used to test IAA production according to Slama et al. [32]. The IAA concentration was estimated using a standard calibration curve using IAA solutions (0 to 10^−3^ M).

#### 2.2.4. Growth on medium without nitrogen

The strains were tested on a WS medium [33] to verify their ability to grow on a medium without nitrogen.

#### 2.2.5. Ammonia production

The ammonia production (NH_3_) capacity was evaluated according to Cherif-Silini et al. [27]. Peptone water was seeded and incubated for 28 ± 2 °C/72 h. The production of ammonia was then revealed by adding 0.5 mL of Nessler's reagent. Color change from yellow to brown indicated ammonia production.

#### 2.2.6. Hydrogen cyanide (HCN) production

The bacterial isolates were inoculated into HCN medium (nutrient agar supplemented with 4.4 g/L of glycine) and incubated at 28 ± 2 °C/4 days. The color change of Whatman paper, placed inside the lid of the plate, impregnated with a developing solution (0.5% picric acid and 2% sodium carbonate) towards red-orange indicated the production of HCN [27].

#### 2.2.7. ACC deaminase production

1-aminocyclopropane-1-carboxylate (ACC) deaminase activity of the bacterial strains was evaluated according to the method of Li et al. [34], using the minimal medium of Dworkin and Foster (DF) free of nitrogen. The cell pellets recovered after culture on LB medium (28 ± 2 °C/48 h) were washed and suspended in DF medium supplemented with ACC (3 mmol/L) as the sole source of nitrogen. After incubation (28 ± 2 °C/24 h), 0.5 mL of supernatant was mixed with 1 mL of ninhydrin reagent. The reaction mixture was heated in a boiling bath for 30 min. Absorbance was measured at 570 nm. Uninoculated DF medium was used as a blank. A bacterial isolate, which produced a darker-colored supernatant than the control, was considered an ACC-utilizing bacterial isolate.

#### 2.2.8. Biofilm production

Biofilm formation was carried out using MSgg medium [35]. 5 µL of the culture of the bacterial strains on LB medium were spotted on MSgg agar (2.5 mM PBS, 100 mM MOPS, 50 µM FeCl_3_, 2 mM MgCl_2_, 50 µM MnCl_2_, 1 µM ZnCl_2_, 2 µM thiamine, 50 mg phenylalanine, 0.5% glycerol, 0.5% glutamate, and 700 µM CaCl_2_, pH 7) incubated at 28 ± 2 °C/72 h. The morphology of the colonies was observed under a magnifying glass for biofilm formation. All PGP activities examined have been performed in triplicate.

#### 2.2.9. Screening of bacterial strains for abiotic stress tolerance capacity

The 24 bacterial strains were evaluated for their ability to tolerate salinity, pH, temperature, and drought. The bacterial cultures (10^8^ cells/mL) were grown on LB broth (Luria–Bertani), supplemented with different concentrations of PEG-6000 (0%, 10%, 20%, and 30%), increasing concentrations of NaCl (0, 200, 400, 600, 800, 1000, and 1200 mM), at different pH values (4, 7, 9, and 11) and incubated at different temperatures (4, 10, 20, 30, 37, 45, and 50 °C) for 48 h. Bacterial growth was assessed by measuring optical density (OD) at 600 nm. Measurements were carried out three times.

#### 2.2.10. Resistance to heavy metals

The tolerance levels of the bacteria to heavy metals (Lead (Pb), Cadmium (Cd), Cobalt (Co) and Mercury (Hg)) were evaluated after seeding by spot 5 µL (10^8^ cells/mL) on LB agar containing different concentrations of metals ranging from 0, 100, 250, 500, and 1000 ppm. The plates were incubated at 28 ± 2 °C/24–72 h.

#### 2.2.11. Tolerance to herbicide and fungicide stresses

The tolerance of bacterial strains to herbicides and fungicides was evaluated by observing their growth on a solid LB medium supplemented with concentrations of the herbicide Tribenuron methyl (TBM) (0.1 µg/mL) and different fungicides (Agrivil 0.5 µL/mL), (Ortiva 0.83 µL/mL), (Dividend 1 µL/mL), (Equation 0.4 µL/mL) and (Tachigazole 1 µL/mL) used according to product recommendations. Spot-seeded plates were incubated at 28 ± 2 °C/24–72 h.

#### 2.2.12. Screening for degradation of polycyclic aromatic hydrocarbons PAHs (Phenanthrene and Bisphenol)

The ability of bacterial strains to degrade and use PAHs as the sole carbon source was evaluated by inoculating 5 µL of bacterial suspension (10^8^ cells/mL) on minimal saline medium (MSM g/L) (Na_2_HPO_4_ 5.8; KH_2_PO_4_ 3; NaCl 0.5; NH_4_Cl 1; MgSO_4_ 0.25, pH of 6.8 ± 0.2). supplemented with phenanthrene and bisphenol (50, 100, 150, 200, 250, and 300 mg/L) [36]. Spot-seeded plates were incubated at 28 ± 2 °C/7 days. The results were expressed qualitatively negative (−) (no growth), or positive (+) (presence of growth) after comparison with the control. All the experiments were conducted in triplicate.

### 2.3. PGP activity of selected strains under osmotic stress

The strains namely ME1, Pa, MR6, and D13, identified as *Providencia vermicola*, *Pantoea agglomerans*, *Pseudomonas knackmussi*, and *Bacillus* sp., respectively, examined for their growth performance under different abiotic stresses (pH 4, 9, temperature 50 °C, NaCl 1000 mM, and PEG 30%) were chosen. They were then evaluated for their PGP activities (phosphate solubilization, IAA, and siderophores production) at different concentrations of PEG-6000 (0, 10, 20, and 30%). The quantitative evaluation was determined according to the methods described previously. The experiments were carried out three times.

### 2.4. Effect of bacterial strains on wheat growth under drought stress

#### 2.4.1. Plant material

Durum wheat seeds of the Bousselam variety (*Triticum durum* L.c.v Bousselam) were obtained from the Technical Institute of Field Crops ITGC (Sétif, Algeria). Seeds were sterilized with 70% ethanol for 2 min, followed by 2% sodium hypochlorite (NaClO_2_) solution for 15 min, then washed several times with sterile distilled water.

#### 2.4.2. *In vitro* germination test

Bacterial inoculum of the four selected strains (ME1, Pa, MR6, D13) were prepared in LB broth and incubated (28 ± 2 °C/48 h) with shaking. The cells were centrifuged at 4000 rpm/20 min at 4 °C. The pellets were washed twice and suspended in sterile saline solution to obtain 10^8^ cells/mL. Germination was carried out in three repetitions and five treatments: Control, ME1, Pa, MR6, D13 in the absence and presence of concentrations of PEG-6000 (10 and 20%). The sterilized seeds were soaked in the bacterial suspensions for 30 min and in distilled water for control. The seeds were then placed in Petri dishes on a double layer of filter paper soaked in 7 mL of sterile distilled water or PEG solution (10 and 20%). Plates were incubated in the dark at 22 ± 2 °C. Germinated seeds were counted after 3, 6, 9, and 11 days. Seeds were considered germinated when the radicle was at least 3 mm long. Four parameters were recorded during this experiment: final germination percentage (FPG) Eq 2, germination rate index (GRI) Eq 3, seedling length vigor index (SLVI) (Eq 4, and seedling weight vigor index (SWVI) Eq 5, calculated as described by [24].

FPG=Number of germinated seeds/Total number of seeds×100 (2)

GRI=G3/3+G6/6+G9/9 (3)

G3, G6, and G9 were the germination percentages at 3, 6, and 9 days.

SLVI=Seedling length (cm)×(%) germination. (4)

SWVI=Seedling weight (mg)×(%) germination. (5)

#### 2.4.3. Pots experiments

Pots experiments were carried out to evaluate *in vivo* the potential of the four drought-tolerant bacteria in alleviating drought stress in wheat. Wheat seeds were sterilized and germinated for 3 days as indicated in the previous section. Ten germinated seeds were planted in plastic pots (ø = 10 cm) (previously sterilized with a solution of sodium hypochlorite NaClO_2)_ filled with 750 g of washed and sterilized sand (180 °C/2 h, 3 successive days). The pots were first amended with ½ Hoagland nutrient solution.

After emergence, the number of seedlings was reduced to 5 seedlings per pot. The experiments were designed with three irrigation regimes: well-watered (100% FC of field water capacity, the amount of water that was required to saturate the soil), moderate water stress (50% FC), and severe water stress (25% FC). Four treatments indicating the type inoculum (ME1, Pa, MR6, D13) and a control without inoculation. Thus, the pots were divided into five groups with five pots per treatment.

Group 1: Negative control: 100% FC, 50% FC, 25% FC.

Group 2: Inoculated with ME1: 100% FC, 50% FC, 25% FC.

Group 3: Inoculated with Pa: 100% FC, 50% FC, 25% FC.

Group 4: Inoculated with MR6: 100% FC, 50% FC, 25% FC.

Group 5: Inoculated with D13: 100% FC, 50% FC, 25% FC.

The pots were organized according to a random plan under a temperature of 16–26 °C, and a photoperiod of 16/8 h. One week later, water stress was applied by maintaining the water content of the pots at 50% and 25% FC. Watering under stress was maintained by measuring the weight of the pots. Two inoculations of the pots were carried out, one at 10 days (3 leaf stage) and the other after 30 days of plant growth. Each pot was inoculated with 1 mL of each bacterial culture (10^8^ cells/mL) and 1 mL of sterile water for the control.

##### 2.4.3.1. Analysis of morphological parameters of the wheat growth

The plants were harvested after 45 days of growth and were washed with water. The lengths of shoots and roots and their fresh weight were measured. The dry weights were measured after drying at 70 °C until constant weight. All morphological parameters were done in triplicate.

##### 2.4.3.2. Quantification of chlorophyll contents

The chlorophyll contents (chl a, b, a+b and carotenoids) of wheat leaves were made according to the protocol described by Cherif-Silini et al. [27]. 0.25 g of leaves from each sample cut into small segments was homogenized in 5 mL of 80% acetone and stored at −20 °C overnight. The organic extract was centrifuged at 14,000 rpm/5 min, and absorbance of the supernatant was measured at 663, 645, and 470 nm to determine chlorophylls a, b, total and carotenoids as shown in Eqs 6–9, respectively.

$$\text{Chl a }\left(\text{mg/g}\right)=\left(\text{12}.{\text{7A}}_{\text{633}}-\text{2}.{\text{69A}}_{\text{645}}\right)$$ (6)

$$\text{Chl b }\left(\text{mg/g}\right)=\left(\text{22}.{\text{9A}}_{\text{645}}-\text{4}.{\text{68A}}_{\text{633}}\right)$$ (7)

Chl a+b (mg/g)=Chl a+Chl b (8)

$$\text{Carotenoids}=\left(\left(\text{1}000{\text{A}}_{\text{47}0}-\text{1}.\text{9Chl a}-\text{63}.\text{14Chl b}\right)/\text{214}\right)$$ (9)

##### 2.4.3.3. Lipid peroxidation

The malondialdehyde (MDA) content, measuring the lipidic peroxidation, was determined by the reaction with thiobarbituric acid (TBA). 0.2 g of leaves cut into very small pieces were macerated in 1 mL of TCA trichloroacetic acid (0.1%) overnight at 4 °C. After centrifugation at (10,000 rpm/5 min) 0.5 mL of the supernatant was mixed with 2 mL of TCA (20%) containing 0.5% TBA. The mixture was heated to 95 °C/30 min, then quickly cooled in an ice bath to stop the reaction. The mixture was centrifuged and the absorbance of the supernatant was measured at 532 nm. The concentration of MDA (nmol/g fresh weight (FW)) was calculated using the extinction coefficient of 155 mM^−1^ cm^−1^ [24].

##### 2.4.3.4. Quantification of proline content

Proline accumulation in leaves and roots was determined by the method described by Saadaoui et al. [37]. Briefly, 50 mg of each sample was homogenized in 1 mL of 40% (v/v) ethanol overnight at 4 °C. After centrifugation at 14,000 rpm/10min, 0.5 mL of supernatant was added to 1 mL of the reaction mixture (ninhydrin 1% (w/v), 60% (v/v) acetic acid) heated to 95 °C/20 min then cooled to room temperature. The centrifuged mixture was measured at 520 nm. The concentration of proline expressed in µg of proline/g of FW was determined using the standard curve.

##### 2.4.3.5. Quantification of total soluble sugars content

The quantification of soluble sugars in wheat leaves and roots was carried out according to the method described by Dubois et al. [38]. Initially, 0.1 g of leaf or root samples were mixed with 3 mL of 80% (v/v) ethanol incubated in the dark at room temperature for 48 hours. Then, the samples were boiled at 80 °C until the ethanol evaporated. The samples are subsequently diluted with 20 mL of distilled water. The absorbance of the reaction mixture containing 0.5 mL of the sample extract, 0.5 mL of 5% phenol, and 2.5 mL of concentrated sulfuric acid was measured at 490 nm. The color intensity was proportional to the concentration of sugars. The concentration of soluble sugars (mg/g FW) was calculated using a standard curve.

##### 2.4.3.6. Quantification of protein content

The protein content was determined on 0.25 g of leaf reduced to a fine powder in a mortar using liquid nitrogen. These samples were then homogenized with 5 mL of phosphate buffer (100 mM, pH 7.5) containing 1 mM EDTA and 0.01% Triton X-100. The soluble protein content (mg/g) was estimated by the technique of Lowry et al. [39] using bovine serum albumin as a protein standard.

##### 2.4.3.7. Antioxidant enzyme assays

Guaiacol peroxidase GPX

GPX was measured according to Kerbab et al. [24], 0.1 mL of enzyme extract was added to 3 mL of reaction mixture (100 mM phosphate buffer pH 6.5, 15 mM guaiacol, and H_2_O_2_ 0.05%). The kinetic evolution of the absorbance at 470 nm was measured for 2 min. Increased absorbance indicated the oxidation of guaiacol. Enzyme activity was calculated using the extinction coefficient of 26.6 mM^−1^ cm^−1^, and expressed in U/min/mg of protein.

Superoxide dismutase SOD

Superoxide dismutase (SOD) activity was assessed using the nitro blue tetrazolium (NBT) method according to Kerbab et al. [24]. 0.1 mL of enzyme extract was added to 3 mL of the reaction mixture (50 mM phosphate buffer at pH 7.5, 75 µM nitro blue tetrazolium chloride (NBT), EDTA, 50 mM sodium carbonate, 2 mM riboflavin). The reaction was exposed to fluorescent light during 15 min. A reaction without enzyme, giving the maximum color, served as a control. The absorbance was measured at 560 nm, and the SOD expressed in U/mg of protein was calculated by the difference between the absorbance of the control and enzyme.

Catalase CAT

To determine the catalase activity, 0.1 mL of enzyme extract was added to 2 mL of reaction mixture (50 mM potassium phosphate buffer at pH 7 and 20 mM H_2_O_2_). The reduction in absorbance was monitored at 240 nm for 2 min. Catalase enzymatic activity was quantified as U/min/mg of protein using the extinction coefficient of 0.036 mM^−1^ cm^−1^ [24].

#### 2.4.4. Survival capacity of bacteria *in vivo*

The survival of bacterial inoculants in wheat plants under water stress was recorded by harvesting 1 g of rhizospheric soil, taken from the surface of the roots, and mixed with 10 mL of sterile physiological water, stirring for 30 min. Decimal dilutions of the samples, up to 10^−6^ were made and 0.1 mL of each dilution was spread on the surface of the LB medium incubated at 28 ± 2 °C/48 h. Detection of endophytic bacteria was also carried out. Roots were surface disinfected by immersion in 70% ethanol/1 min, then in 2% sodium hypochlorite/30 min and rinsed several times with sterile distilled water. To check sterilization, the final wash water was spread onto LB agar plates and incubated at 28 ± 2 °C/48 h. One g of sterilized roots was mixed and crushed in 10 mL of sterile physiological water. Bacterial counts were carried out as before. The survival rate of bacteria, expressed in CFU/g of soil or CFU/g of roots was determined by duplicate experiments.

#### 2.4.5. Root colonization ability (RCA)

The wheat seeds were sterilized and immersed for 30 min in bacterial suspensions containing 10^8^ cells/mL of ME1, Pa, MR6, and D13 strains. Seeds germinated in Petri plates for 3 days were sown in tubes containing 10 mL of MS agar medium [40] containing PEG-6000 concentrations (0 to 10 and 20%). The experiments were performed three times and incubated in a culture chamber for 11 days at 25 °C. Untreated seeds were used as a control. The ability of the strains to colonize the surface of plant roots was studied visually by microscopic analysis using the triphenyl tetrazolium chloride (TTC) procedure according to the protocol of Cherif-Silini et al. [27]. Wheat roots were incubated in TTC solution (0.15% TTC in 0.06 M PBS, pH 6.8) for 2 hours in the dark. The development of pink areas indicated the presence of large amounts of bacteria reducing colorless TTC.

### 2.5. Statistical analysis

All the experiments were repeated three times and the results were expressed as mean ± standard error of the mean. The data were analyzed using GraphPad Prism 8. One-way ANOVA and two-way ANOVA were used. The groups were compared using a Tukey's HSD test to analyze the data to find whether there was significant effect of the treatment compared to the control sample. The level of significance used for all statistical tests was 5% (p < 0.05).

## 3. Results

### 3.1. In vitro screening of bacteria for plant growth promoting activities and tolerance to abiotic stresses

#### 3.1.1. *In vitro* screening of PGP activities

The study investigated the PGP characteristics of 24 bacterial isolates, revealing diverse activities. All strains exhibited phosphate solubilization, with varying rates; strains B10, OR15, RK2, and Pa showed the highest production (up to 180 µg/mL) (Figure S1 A). Auxin (IAA) production varied among strains, with maximum levels in Pa (180 µg/mL) and ME1 (130 µg/mL) (Figure S1 B). Siderophores production occurred in most strains, with the highest production in MR6 (68%), MN8 (58%), and Pa (50%) strains (Figure S1 C). ACC-deaminase activity was widespread, with Pa and OR15 exhibiting the best activity (Figure S1 D). Most strains demonstrated nitrogen fixation and ammonia production, while only MR8, MR4, MN8, and MN6 produced hydrogen cyanide. Biofilm formation was evident in the majority of strains, with FZB, BR5, D13, Pa, RB13, and OR15 showing maximum production (Figure S2).

#### 3.1.2. Screening of bacterial strains for abiotic stress tolerance capacity

The results of the stress tolerance of the 24 bacterial strains offered a comprehensive overview of the strains' responses to the different abiotic stresses. Increasing PEG concentrations negatively impacted bacterial growth, revealing a pronounced osmotic effect. Strains displayed varying degrees of tolerance at PEG concentrations between 10% and 20%. Notably, strains D13, RB13, Pa, ME1, and MR6 exhibited growth even at 30% PEG, demonstrating tolerance to osmotic stress (Figure S3).

Bacterial growth was optimal in the absence of NaCl (0 mM) but declined significantly with increasing NaCl concentrations. Strains exhibited tolerance up to 1000 mM NaCl, with only a select few (RB13, BR5, Pa, ME1, MR6, MN8, MN6, MR1) tolerated 1200 mM NaCl, indicating a high degree of salt stress tolerance (Supplementary Figure S4).

All strains were found to be neutrophilic, preferring optimal growth at pH 7, but showed sensitivity to both acidic and alkaline conditions. Strains BR5, RB13, and OR15 demonstrated tolerance by growing at pH 4, while most strains-maintained growth in the alkaline range of pH 9 to 11 (Figure S5). Bacterial growth was observed through a wide temperature range (20 °C to 37 °C), with the optimal growth temperature around 30 °C. Some strains, such as Pa and MN6, exhibited the best growth at 37 °C. Most strains demonstrated a notable growth at 45 °C but were very weak at 50 °C (Figure S6).

#### 3.1.3. Heavy metal tolerance capacity

The results of the impact of heavy metals (lead, cadmium, cobalt and mercury) on bacterial growth were shown in Figure S7 A. According to the results, the strains seemed to tolerate Pb well up to 1000 ppm. However, they were sensitive to Co, Cd and Hg and could only tolerate low concentrations (50 and 100 ppm). Compared to all the strains, the *Bacillus* (RB13, D12, D13, BR5, B28, B25, B22, B16, and B6) showed a high tolerance to Cd.

#### 3.1.4. Degradation capacity of polycyclic aromatic hydrocarbons HAP (phenanthrene and bisphenol)

The ability of the 24 bacterial strains to degrade phenanthrene and bisphenol at different concentrations (0, 100, 150, 200, 250, and 300 mg/L) was evaluated by observing their growth on LB agar (Figure S7 B). The results indicated that the majority of *Bacillus* strains degraded phenanthrene. However, they were more sensitive to bisphenol and grew at low concentrations (100 or 150 mg/L). The other bacteria presented mixed results and generally tolerated only low concentrations of phenanthrene or bisphenol with the exception of MR1, MR6 and MR8 strains, which showed an ability to degrade high concentrations of bisphenol.

#### 3.1.5. Herbicides and fungicides tolerance capacity

The tolerance of bacterial strains to herbicides (Tribenuron methyl TBM) and fungicides (Ortiva, Divedend, Tachigazole, Agrivil and Equation) was evaluated by observing their growth on LB agar supplemented with concentrations of herbicides and fungicides. As shown in Supplementary Figure S7 C. All strains were tolerant to herbicides, indicating the absence of herbicide toxicity on bacterial growth. In addition, all bacterial strains were tolerant to Ortiva and the majority could grow in the presence of the equation, Tachigazole and Agrivil. Except for B6, B10, B28, B16, and B25 ME1 strains, the rest were Dividend resistant.

### 3.2. PGP activities of selected bacteria under osmotic stress

**Figure 1.**
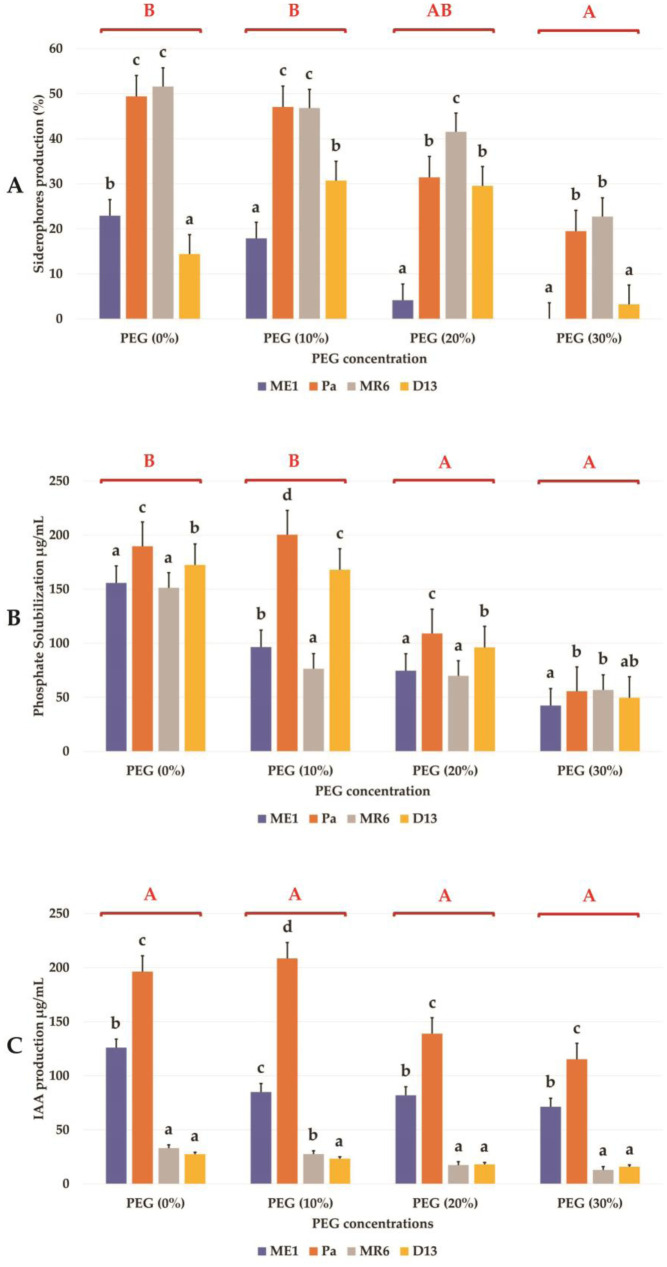
Effect of varying PEG concentrations (0, 10, 20 and 30%) on PGP activities of the bacterial strains (ME1, Pa, MR6, and D13), (A) siderophores production (%), (B) Phosphate solubilization de (µg/mL) and (C) IAA production (µg/mL). Values are means ± standard error of three replicates. Lowercase letters (a, b, c) indicate significant differences (p < 0.05) between the control and the different isolates. The capital letters (A, B, C) indicate the different water stress levels (0, 10, 20, and 30% of PEG). Two-way ANOVA setting followed by Tukey's multiple comparison post-test are used to identify the differences between the different drought stress treatments.

Four strains (ME1, Pa, MR6, and D13) were chosen among the 24 strains based on their performance under extreme abiotic stresses conditions (Figure S8). These strains were evaluated for their PGP activities, at varying concentrations of PEG (0, 10, 20, and 30%). Siderophore production exhibited variations in response to increasing PEG concentrations (Figure 1A). Increasing PEG concentration reduced siderophore production, but appreciable levels were maintained at 10% and 20% PEG. Best siderophore production was observed for strains Pa and MR6 without stress (0% PEG). With the increase in the PEG concentrations, a progressive decrease in the concentration of soluble phosphates was observed for the majority of strains with the exception of Pa strain where a maximum of solubilization was noted at 10% PEG (200 µg/mL). The solubilization of phosphate at 20% PEG recorded appreciable rates (120 µg/mL) (90 µg/mL) with the Pa and D13 strains respectively (Figure 1B). IAA production also showed reductions in response to increasing PEG concentrations for ME1, MR6 and D13 strains (Figure 1 C). However, the Pa strain showed better production (210 µg/mL) at 10% PEG. This strain maintained appreciable levels (140 µg/mL) and (120 µg/mL) at 20% and 30% PEG respectively. Regarding the ME1 strain, the production of IAA decreased, but an appreciable production of 70 µg/mL was observed at 30% PEG (Figure 1 C). Our findings emphasize the influence of abiotic stress on the PGP capabilities of these strains and provide insights into their potential applications in stressed environments.

### 3.3. Effects of bacterial strains on wheat growth under water stress

#### 3.3.1. Seed germination test

**Figure 2.**
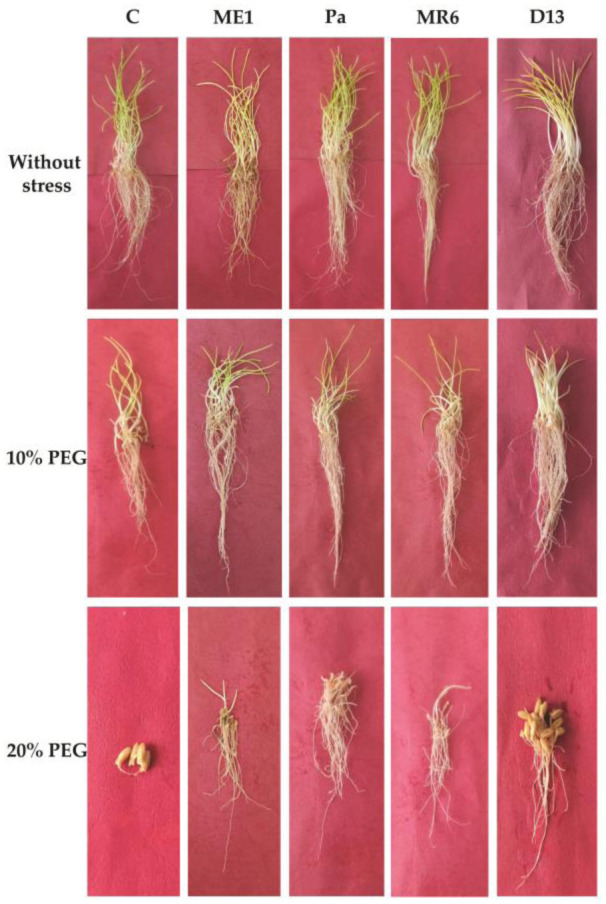
Effect of bacterial isolates (ME1, Pa, MR6 et D13) on, *in vitro*, seedling wheat germination under three drought stress treatments (0, 10, and 20% of PEG) compared to control without stress.

**Figure 3.**
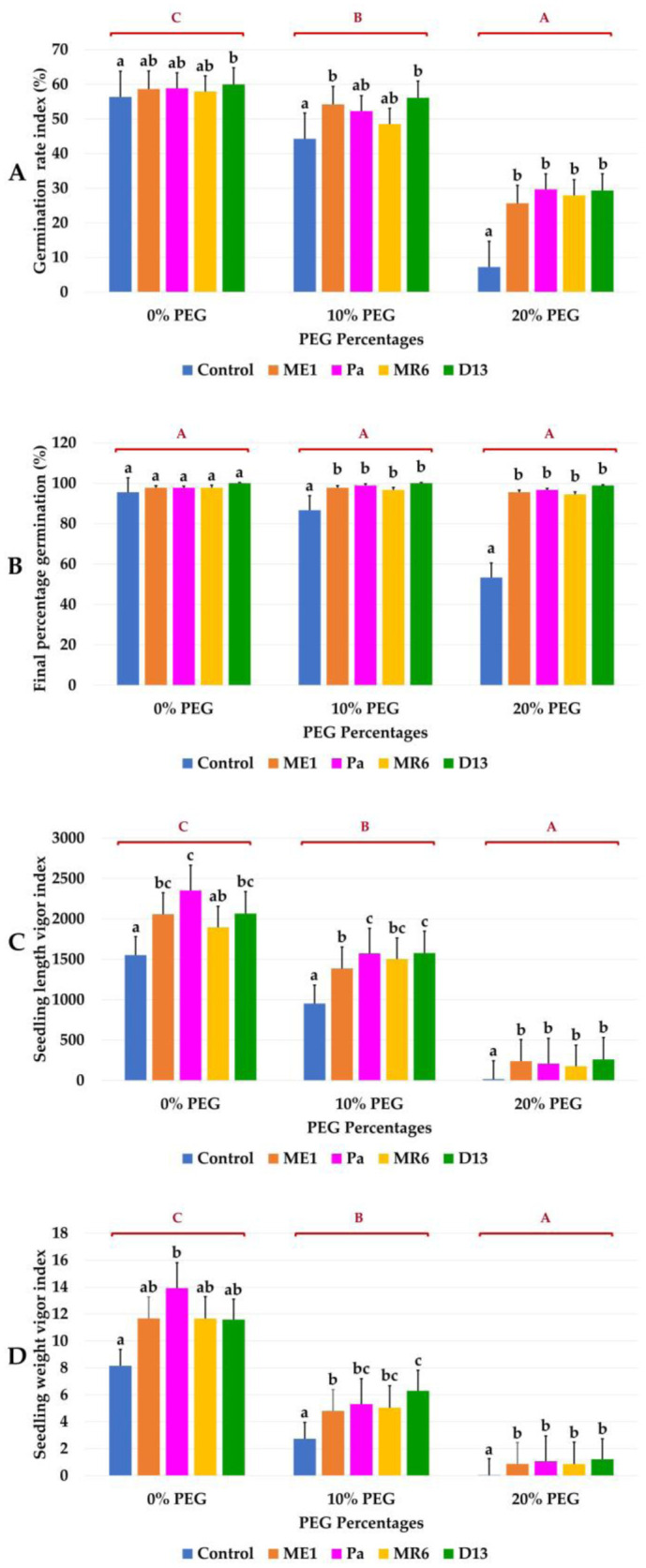
Effect of bacterial isolates (ME1, Pa, MR6, D13) on (A) Germination rate index GRI (%), (B) Percentage of final germination PGF (%), (C) Seedlings length vigor index SLVI, and (D) Seedling weight vigor index of seed wheat under three drought stress treatments (0, 10, and 20% of PEG). Values are means ± standard error of three replicates. Lowercase letters (a, b, c) indicate significant differences (p < 0.05) between the control and the different bacterial isolates. The capital letters (A, B, C) indicate the different water stress levels (0, 10, and 20% of PEG). Two-way ANOVA setting followed by Tukey's multiple comparison post-test are used to identify the differences between the different drought stress treatments.

The study evaluated the influence of bacterial strains on wheat germination under drought stress. Germination parameters such as final germination percentage (FPG), germination rate index (GRI), seedling length vigor index (SLVI), and seedling weight index (SWVI) were assessed and were presented in Figures 2 and 3. Seed germination parameters were significantly reduced under drought stress (20% PEG). Osmotic stress adversely affected the germination rate index (GRI), showing a low level of 7.22% at 20% PEG (Figure 3A). The PGF had not reached more than 53.33% in uninoculated controls (Figure 3B). The application of bacterial strains improved germination parameters under PEG stress conditions. The D13 strain exhibited remarkable tolerance, achieving 100% PFG under 10% and 20% PEG (Figure 3B). Bacterial inoculation significantly improved GRI under stress conditions, compared to uninoculated controls (Figure 3A).

Seedling length vigor index (SLVI) and seedling weight index (SWVI), representing seed activity and durability, were significantly reduced under 20% PEG-induced osmotic stress. Bacterial inoculation led to a substantial improvement in these indices even under stress conditions (Figure 3C and D). The positive impact of bacterial strains, in enhancing wheat germination and seedling vigor under normal and drought stress suggested the potential application of these bacterial strains for improving crop performance.

#### 3.3.2. Pots experiments

##### 3.3.2.1. Morphological parameters

**Figure 4.**
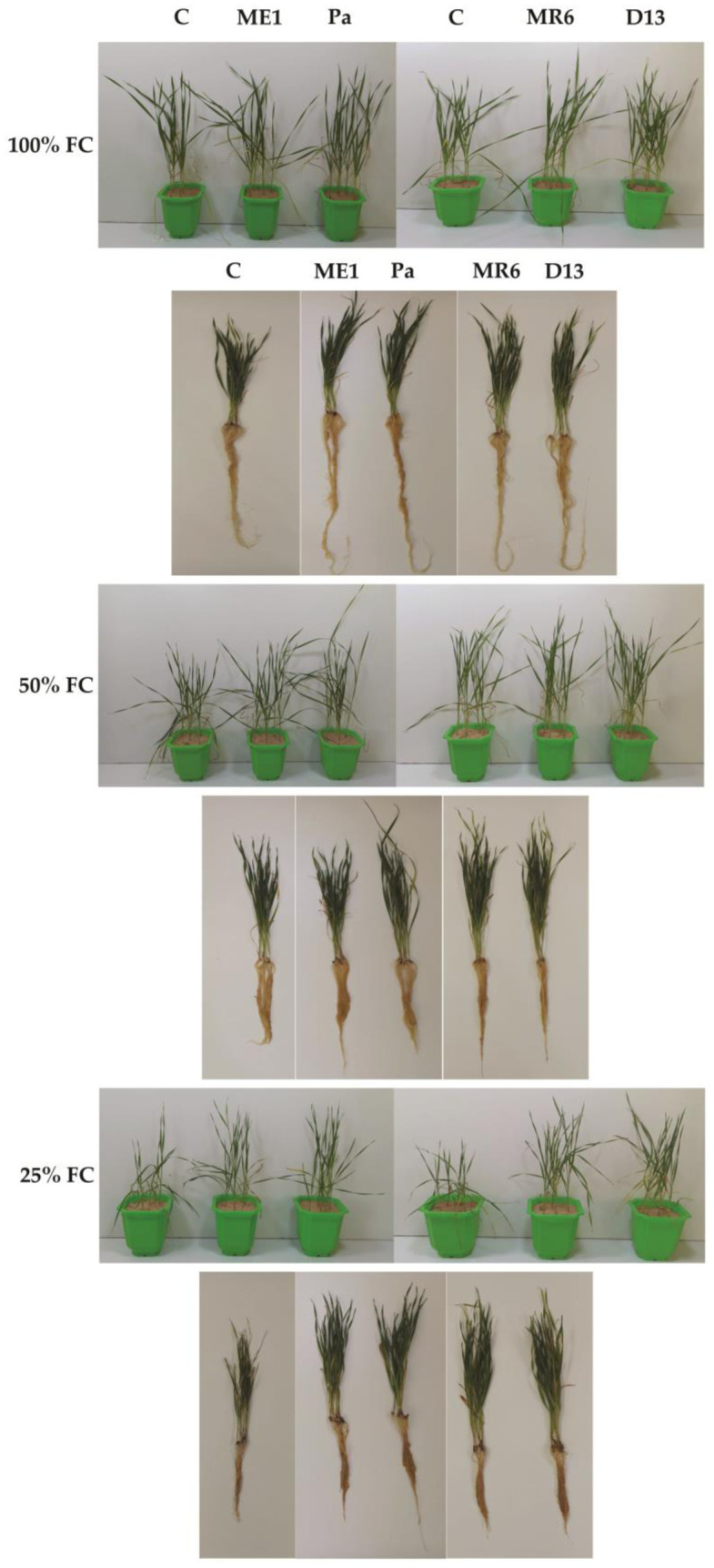
In planta evaluation of bacterial isolates (ME1, Pa, MR6 et D13) compared with control (non-inoculated) on wheat seedlings and their root systems under three drought stress treatments; well-watered (100% FC), moderate stress (50% FC), and severe stress (25% FC).

**Figure 5.**
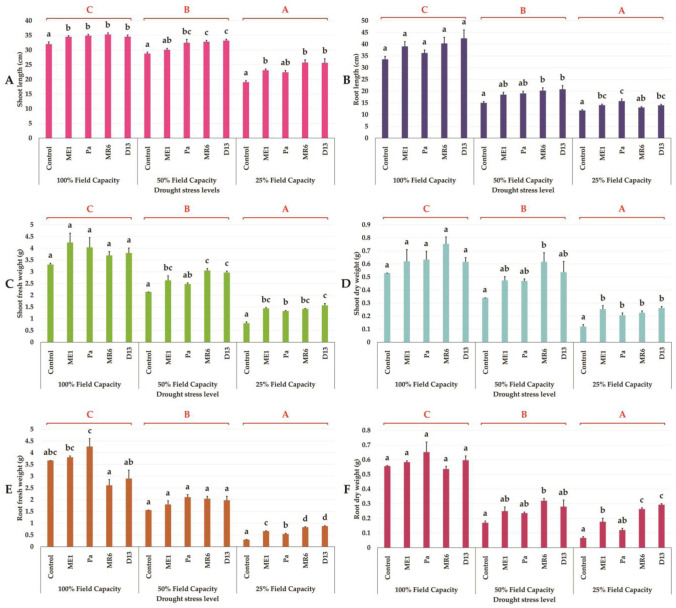
Effect of bacterial inoculation on (A, B) shoot and root length (cm), (C, D) shoot and root fresh weight (g), and (E, F) shoot and root dry weight of wheat plants under three drought stress treatments under three drought stress treatments; well-watered (100% FC), moderate stress (50% FC) and severe stress (25% FC). Values are means ± standard error of three replicates. Lowercase letters (a, b, c) indicate significant differences (p < 0.05) between the control and the different bacterial isolates. The capital letters (A, B, C) indicate the different water stress levels (100, 50, and 25% FC). Two-way ANOVA setting followed by Tukey's multiple comparison post-test are used to identify the differences between the different drought stress treatments.

The experiment revealed a significant decrease in various wheat plant growth parameters under escalating water stress (Figures 4 and 5). Moderate water stress (50% FC) led to important reductions in shoot length (SL), root length (RL) (Figure 5A and B), shoot fresh weight (SFW), shoot dry weight (SDW) (Figure 5C and D), root fresh weight (RFW), and root dry weight (RDW) (Figure 5E and F). while severe water stress (25% FC) resulted in even more pronounced declines. Inoculation with the bacterial strains (Pa, ME1, MR6, and D13) demonstrated a significant positive impact on these growth parameters under both stress and non-stress conditions. Notably, D13 and MR6 strains exhibited superior efficacy in enhancing plant growth, particularly under moderate water stress conditions. The positive effects of inoculation were evident in shoot and root characteristics, with significant increases in fresh and dry weights observed. Overall, the results highlighted the potential of bacterial strains to mitigate the adverse effects of water stress on plant growth.

##### 3.3.2.2. Chlorophyll content

**Figure 6.**
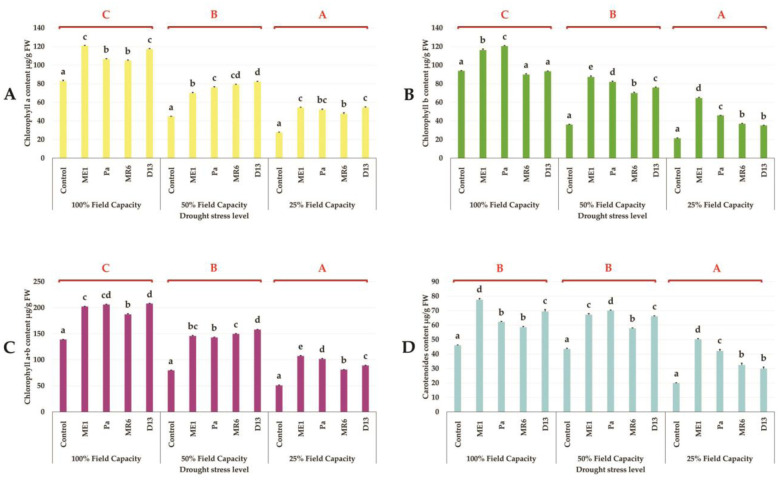
Effect of bacteria isolates on (A) Chlorophyll a (µg/g Fresh Weight), (B) Chlorophyll b (µg/g FW), (C) Chlorophyll a+b (µg/g FW) and (D) Carotenoids (µg/g FW) of wheat plant under three drought stress treatments; well-watered (100% FC), moderate stress (50% FC) and severe stress (25% FC). Values are means ± standard error of three replicates. Lowercase letters (a, b, c) indicate significant differences (p < 0.05) between the control and the different bacterial isolates. The capital letters (A, B, C) indicate the different water stress levels (100, 50 and 25% FC). Two-way ANOVA setting followed by Tukey's multiple comparison post-test are used to identify the differences between the different drought stress treatments.

Chlorophyll pigment contents showed that drought stress led to a significant decrease (p ≤ 0.05) in the levels of Chl a, Chl b, and Chl a+b and carotenoids compared to non-inoculated controls. Water stress (50% and 25% FC) presented very reduced chlorophyll levels, more marked in severe water stress (25% FC). Whereas, inoculation of wheat plants with the bacterial strains significantly increased the content of chlorophyll pigments under normal and stressed conditions. All bacterial inoculations significantly increased the chlorophyll content compared to the non-inoculated control under water stress (50 and 25% FC) (Figure 6).

##### 3.3.2.3. Proline content

**Figure 7.**
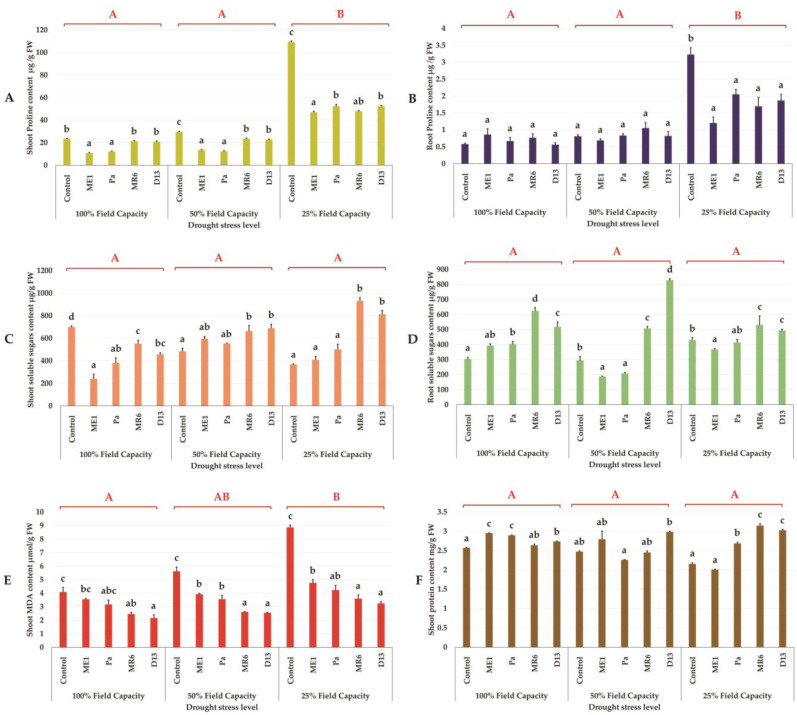
Effect of bacterial inoculation on (A, B) shoot and root proline content (µg/g FW), (C, D) shoot and root total soluble sugars content (mg/g FW), (E) shoot malondialdehyde (MDA) content (µM/g FW) and shoot protein content (mg/g FW) of wheat plants under three drought stress treatments; well-watered (100% FC), moderate stress (50% FC) and severe stress (25% FC). Values are means ± standard error of three replicates. Lowercase letters (a, b, c) indicate significant differences (p < 0.05) between the control and the different bacterial isolates. The capital letters (A, B, C) indicate the different water stress levels (100, 50 and 25% FC). Two-way ANOVA setting followed by Tukey's multiple comparison post-test are used to identify the differences between the different drought stress treatments.

The analysis of the results relating to the concentration of foliar and root proline, under normal irrigation conditions (100% FC) and in the presence of various levels of water stress, revealed a significant increase only during severe water stress (25% FC) (Figure 7A and B). inoculation by different bacterial isolates on proline content was found to be negligible under normal irrigation conditions and in the presence of moderate water stress. However, this inoculation resulted in a significant decrease in proline accumulation under severe drought stress (25% FC). Proline plays a crucial role as a compatible solute for osmoregulation. Its reduction observed under severe water stress suggested a reduction in oxidative stress in inoculated plants compared to non-inoculated plants. These results indicated that inoculation enhanced plant resistance to water stress by modulating proline levels.

##### 3.3.2.4. Total sugars content

Our results indicated that unlike proline, water stress did not have a statistically significant impact on the concentration of total sugars in roots and leaves (Figure 7C and D). However, the effect of inoculation on the accumulation of these sugars varied depending on the bacterial isolates and the level of water stress. We observed a more pronounced trend with isolates MR6 and D13 under moderate and severe drought stress, respectively. Interestingly, bacterial inoculation had a notable effect on plants subjected to severe water stress (25% of field capacity). In particular, inoculation with strains MR6 and D13 resulted in a significant increase in leaf sugar content compared to other non-inoculated control treatments. This observation suggests a potential role of strains MR6 and D13 in the regulation of sugar metabolism under conditions of severe water stress. This is one of the defense strategies of bacterial strains to make plants more tolerant to drought. Total soluble sugars also serve as osmolytes in plant cells, providing protection against osmotic stress.

##### 3.3.2.5. Lipid peroxidation

We evaluated MDA content under water stress and non-stress conditions, with or without inoculation (Figure 7 E). Results showed an increase in MDA content under water stress. The highest levels of MDA were observed in uninoculated controls under severe drought stress. Inoculated plants exhibited a lower amount of MDA at all levels of water stress. The inoculation of strains D13 and MR6 significantly reduced MDA levels, indicating its positive effect, especially for moderate and severe stress levels. These results indicated that inoculation played a protective role in wheat plants, protected plant cellular homeostasis and mitigating the negative effects of drought stress.

##### 3.3.2.6. Protein content

Our results revealed that leaf protein content did not show significant difference between normal irrigation (100% FC), moderate stress (50% FC) and severe stress (25% FC) conditions (Figure 7E). Furthermore, no notable variation was observed between inoculated and non-inoculated plants under 100% FC irrigation conditions, indicating stability in protein content regardless of bacterial inoculation. However, a positive effect of bacterial inoculation on protein contents was observed in plants subjected to severe stress (25% FC), in particular with strains Pa, MR6 and D13. The observation of a positive effect of bacterial inoculation on protein levels in plants subjected to severe stress can be attributed to the capacity of inoculated bacteria to stimulate defense and adaptation mechanisms, thus helping plants to better cope with environmental stress.

##### 3.3.2.7. Effects on antioxidant enzymes activities

**Figure 8.**
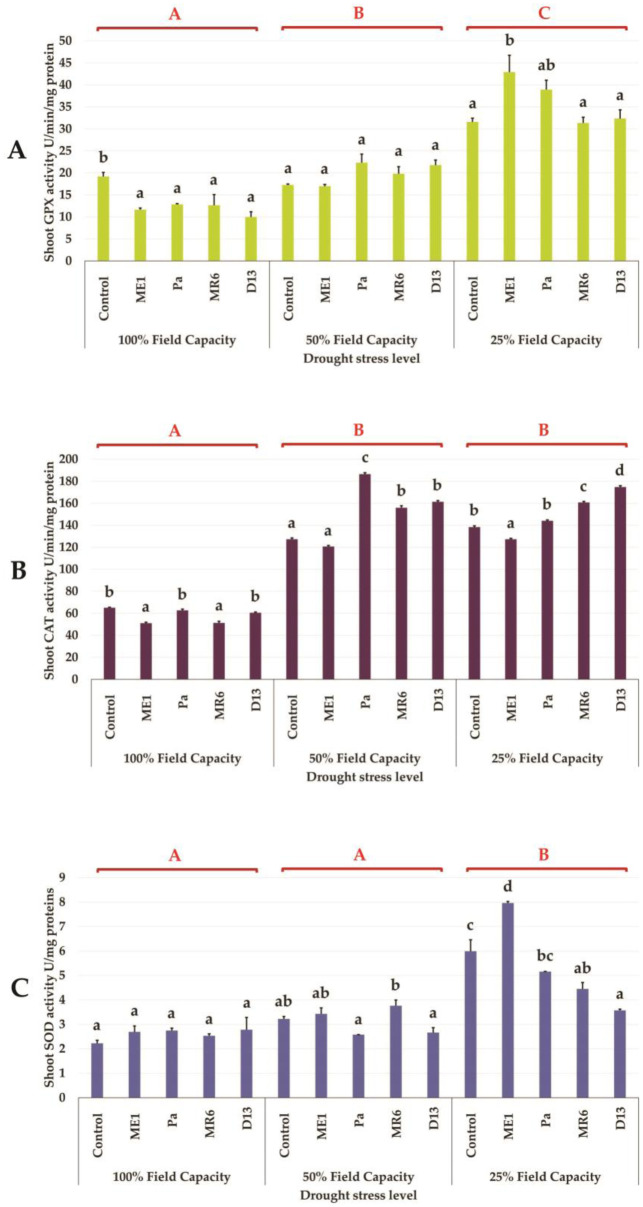
Effect of bacterial isolates on (A) guaiacol peroxidase (GPX) content (U/min/mg of protein), (B) catalase (CAT) content (U/min/mg of protein) (C) superoxide dismutase (SOD) content (U/mg of protein) of wheat plants under three drought stress treatments; well-watered (100% FC), moderate stress (50% FC) and severe stress (25% FC). Values are means ± standard error of three replicates. Lowercase letters (a, b, c) indicate significant differences (p < 0.05) between the control and the different bacterial isolates. The capital letters (A, B, C) indicate the different water stress levels (100, 50 and 25% FC). Two-way ANOVA setting followed by Tukey's multiple comparison post-test are used to identify the differences between the different drought stress treatments.

The activity of antioxidant enzymes such as GPX, CAT, and SOD in inoculated or uninoculated plants increased proportionally to the intensity of water stress (Figure 8A, B, and C). This increase was more significant at a severe stress level (25% FC), mainly observed for GPX and SOD (Figure 8A and C). Regarding CAT, stimulation of its activity was observed at a level of 50% FC (Figure 8B). The positive effect of bacterial inoculation on the activity of antioxidant enzymes varied depending on the bacterial isolates used. At 25% FC, the enzymatic activity of GPX was more intense in the presence of isolates ME1 and Pa, while that of CAT was stimulated by MR6 and D13. Regarding SOD, a significant increase was observed only in the presence of isolate ME1 (Figure 8C).

##### 3.3.2.8. Effect of drought stress on survival and colonization of rhizospheric and endophytic bacterial density

**Figure 9.**
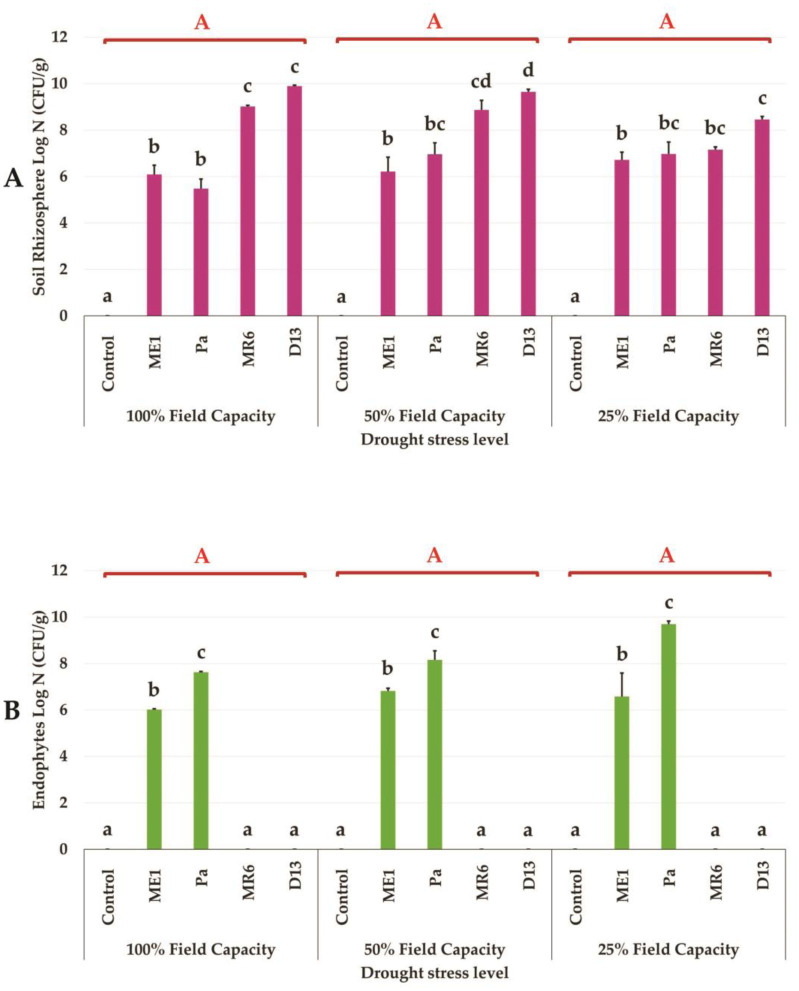
Survival of bacteria, ME1, Pa, MR6, and D13 (Log N (CFU/g)), in (A) rhizosphere and (B) roots of wheat plants grown under three drought stress treatments; well-watered (100% FC), moderate stress (50% FC) and severe stress (25% FC). Values are means ± standard error of three replicates. Lowercase letters (a, b, c) indicate significant differences (p < 0.05) between the control and the different bacterial isolates. The capital letters (A, B, C) indicate the different water stress levels (100, 50, and 25% FC). Two-way ANOVA setting followed by Tukey's multiple comparison post-test are used to identify the differences between the different drought stress treatments.

The results of bacterial survival in the rhizosphere and root of the wheat plant were shown in Figure 9. The inoculated strains demonstrated significant colonization of the rhizosphere and roots of wheat under normal conditions and drought stress. The bacterial survival rate in the rhizosphere of wheat plants was found to be significant, reaching values of approximately 10^8^–10^9^ UFC/g soil for the rhizosphere strains MR6 and D13, and maintaining notable stability under normal and moderate stress. A slight decrease in bacterial numbers was observed under severe stress conditions, with counts around 10^7^ and 10^8^ UFC/g for MR6 and D13 respectively (Figure 9A). The enumerations of ME1 and Pa strains in the rhizosphere increased proportionally to the intensity of the stress, going from 10^5^ UFC/g for Pa and from 10^6^ UFC/g for ME1, under normal conditions to 10^7^ UFC/g under stressed conditions (Figure 9A). On the other hand, their presence in the roots, due to their endophytic nature, showed a more marked increase from 10^6^ UFC/g of roots under normal conditions to 10^7^ UFC/g under stressed conditions for strain ME1. The Pa strain showed better colonization capacity under severe stress (25% FC), with an increase in bacterial enumerations from 10^7^ UFC/g under normal conditions to 10^9^ UFC/g under stressed conditions (Figure 9B). These observations support the effective nature of the strain Pa under various stresses.

**Figure 10.**
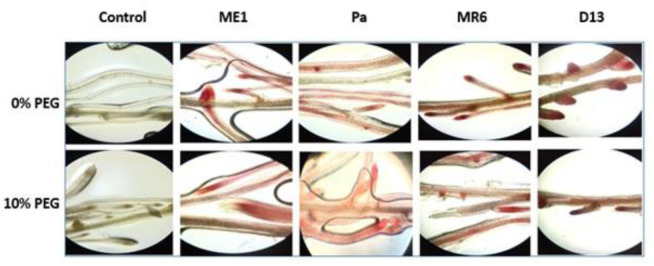
Microscopic examination of plant roots and bacterial strains (ME1, Pa, MR6 et D13) colonizing roots under osmotic stress (0 and 10% PEG) compared to non-inoculated roots.

The ability of the strains to colonize the plant surface was indicated by microscopic examination. The wheat roots of the different treatments cultivated on MS in the presence of different concentrations of PEG and incubated in the TTC solution showed the clear presence of pink zones on the surface of the roots (Figure 10). These results attested to the presence of enormous quantities of bacterial strains colonizing the surface of wheat roots.

## 4. Discussion

Water stress is a major agricultural challenge globally that affects the growth, morphology, physiology, and biochemistry of plants and significantly limits the production of crops especially in arid and semi-arid zones [13]. Over the past decade, researchers have increased the understanding of the role of PGPB in alleviating water stress in plants. This research development highlights the promising potential of PGPB as a strategic tool for managing and improving plant tolerance to water stress. PGPB act by modulating the physiological response of plants to drought. Given the variations in ecological characteristics and abiotic circumstances of the soil, it is imperative to carefully select beneficial microorganisms adapted to these specific conditions to ensure their successful application in the field.

Our objective was to evaluate and select PGPB demonstrating tolerance to various abiotic stresses, while exhibiting multiple beneficial activities. This approach is of particular importance in the context of meeting the nutritional needs of plants grown in arid, semi-arid, polluted, or poor-quality soils. Thus, this research made it possible to ex-amine the capacity of 24 bacterial isolates from various ecosystems to resist various abiotic stresses. These bacteria were tested to evaluate their PGP characteristics, including their ability to produce IAA, solubilize phosphate, produce ACC deaminase, siderophores, ammonia, HCN, fix nitrogen, and produce biofilm [12],[20]. Additionally, their ability to grow under abiotic stress conditions such as salinity, drought, pH, and temperature was assessed. These bacteria have also demonstrated tolerance to heavy metals, pollutants, fungicides, and herbicides, making them suitable for use in varied environmental conditions. Previous research indicated that bacteria with multiple beneficial traits and resistance to various stresses are preferable to those with just one. This finding suggested that the versatility of bacteria, their ability to meet various needs and resist different environmental constraints, makes them more effective in their interactions with plants [41],[42].

Screening of the 24 PGPB strains resulted in the selection of four bacteria (ME1, Pa, MR6, and D13) exhibiting diverse PGP activities and notable tolerance to different abiotic stresses. These strains were then subjected to evaluation of their PGP properties, including auxin (IAA) production, siderophore synthesis, and phosphate solubilization under osmotic stress conditions (0%, 20% and 30% PEG). IAA production decreases depending on the intensity of the drought. However, the Pa strain presents a very high concentration of IAA at 10% PEG. Overall, strains ME1, Pa, MR6, and D13 maintained IAA synthesis even at a high concentration of 30% PEG. IAA, essential for cell division, promotes the development of lateral roots, thereby increasing the absorptive surface area and improving the ability of plants to take up water and nutrients under water stress conditions [43],[44]. Un-der similar stress conditions, phosphate solubilization was observed in all strains, with maximum solubilization noted in the Pa strain at 10% PEG. Phosphorus, as an essential macro element, participates in virtually all metabolic functions crucial for plant growth [44]. The selected strains (ME1, Pa, MR6, D13) retained the capacity to produce siderophores even under conditions of osmotic stress. Strains Pa and MR6 stand out as the most efficient producers of siderophores in the presence of PEG. Siderophores play a crucial role in drought conditions due to their iron-chelating mechanisms, thereby improving the availability of this essential nutrient to plants [45],[46].

Seed germination is a crucial phase of the plant life cycle, significantly influencing their further development [10]. The study demonstrated that the application of PEG induces osmotic stress, reducing wheat germination, especially to 20%. Suppression of germination due to drought has been observed in various crops, such as wheat (*Triticum aestivum* L.) [19],[47], maize (*Zea mays* L.) [25], and barley (*Hordeum vulgare* L.) [16]. Osmotolerant bacteria (ME1, Pa, MR6, D13) significantly improve germination in the presence of PEG, highlighting their effectiveness [37],[48]. These strains, due to their osmotolerance, stimulate the synthesis of auxins, promoting cell division and cell elongation despite water stress. Increased production of growth hormones stimulates the activity of enzymes like α-amylase, improving starch absorption and promoting germination [49]. The study confirmed the advantages of using osmotolerant strains producing high concentrations of phytohormones, notably auxin (IAA), on wheat germination under water stress conditions [49]. Previous research on cotton has also shown similar improvements with IAA [50]. Furthermore, the treatment of pigeon pea seeds (*Cajanus cajan* L.) by *P*. *aeruginosa* and *B*. *megaterium*, producing both gibberellic acid and IAA had a positive impact on germination, especially in the presence of water stress [11]. Strains producing exopolysaccharides (EPS), notably strain D13, alleviate osmotic stress, improving germination. These results highlighted the important role of selected bacterial strains in improving germination, particularly under conditions of water stress, due to their osmotolerance, increased synthesis of auxins and production of exopolysaccharides [19],[21].

The pot study evaluated the impact of inoculation of bacterial strains on the growth of wheat plants under different levels of water stress. Stress led to a significant reduction in morphological parameters, especially under conditions of severe stress (25% FC). This decrease is mainly attributable to a deliberate reduction in cell proliferation, aimed at optimizing nutrient and water use. Water stress also led to a decrease in turgor, hindering cell elongation and expansion [9],[20]. Additionally, restriction of aboveground growth may also result indirectly from the closure of stomata, thereby reducing nutrient uptake and photosynthesis rates. Under stress, cells redirect their energy towards defense mechanisms, thereby modifying the cell growth pattern [20]. Several studies have also examined the impact of water stress on the physiological and morphological characteristics of wheat [9],[12],[19],[51].

Inoculation with the four bacterial strains stimulated plant growth, improving the uptake of nutrients from the soil, and making them readily available to plants, even in the presence of stress [13]. Strains producing ACC deaminase alleviated the negative effects of drought stress, reducing ethylene concentration and improving stress tolerance [9]. Ethylene, in high concentrations during stress, can inhibit plant growth, affecting roots, stems, and leaves [9],[20].

Furthermore, biofilm formation by rhizospheric bacteria, with the production of exopolysaccharides, enhanced water stress tolerance by promoting soil aggregation and efficient nutrient uptake [19],[47]. Finally, bacterial inoculation, particularly with strains producing ACC deaminase and promoting biofilm formation, represents a promising strategy for improving plant tolerance to water stress.

Evaluation of chlorophyll content in plants under water stress reveals a significant decrease in chlorophyll pigments [12]. Severe stress conditions accentuate this decline, recording a reduction of 200.94%, 341.24%, 173.79%, and 130.97% for chl a, b, total, and carotenoids respectively compared to non-stress conditions (100% FC). Intense water stress disrupts the photosynthetic machinery, leading to disruption of photosynthesis manifested by a simultaneous decrease in photosynthetic activity, stomatal conductance, and CO2 concentration. Alterations in chloroplasts reduce their photosynthetic capacity [51]–[53]. However, the application of treatments specific and inoculation of PGPB strains showed positive results in increasing chlorophyll levels, thus promoting stress tolerance. These PGPB strains act by improving the absorption of nutrients, particularly nitrogen, essential for chlorophyll [24],[53]. Studies highlighted the association between increased drought tolerance in plants, preservation of chlorophyll levels, and inoculation of PGPB strains. The specific role of certain PGPB such as *Beijerinckia fluminensis* BFC33 [44], *Pseudomonas* sp. and *Serratia marcescens* [46] *Bacillus* sp., *Azospirillum lipoferum* [12] was emphasized in the direct regulation of wheat plant physiology. Additionally, inoculation led to improved photosynthetic pigment concentration and photochemical efficiency, increasing plant productivity under different levels of water stress.

In the presence of water stress, plants react by producing reactive compounds, causing oxidative damage to plant tissues [13]. Malondialdehyde (MDA) is often used as an indicator of lipid peroxidation, revealing a significant increase in its concentration during moderate (50% FC) and severe (25% FC) stress in untreated wheat plants. However, inoculation with bacterial strains (ME1, Pa, MR6, and D13) significantly reduced MDA levels, suggesting attenuation of membrane damage. These results indicated that plants treated with these strains experienced less oxidative stress, confirming previous studies demonstrating the maintenance of cellular redox potential by bacteria [54]. Similar research has shown that bacterial inoculation can improve plant biomass and reduce MDA concentrations under stress, highlighting the beneficial role of bacteria in protecting plants from the detrimental effects of water stress [55].

Osmotic adjustment, characterized by the accumulation of osmoprotectants such as proline, is a fundamental adaptation that improves plant survival during times of stress, particularly drought stress. Proline serves a variety of functions, including maintaining cellular turgor, stabilizing subcellular components, and serving as a signaling molecule [56]. Uninoculated plants showed a significant increase in proline under stress, while bacterial inoculation reduced this accumulation, thereby mitigating the impact of water stress. Studies, such as those by Kasim et al. [51], demonstrated that bacterial inoculation reduced the harmful effects of water stress, with less accumulation of proline. The association with bacteria appeared to promote resistance to water stress, offering prospects for improving plant tolerance to harsh environmental conditions.

Total soluble sugars act as osmolytes, protecting plant cells from osmotic stress by preserving cellular interactions during dehydration. Bacterial inoculation increases sugars in wheat plants under water stress, with variations related to the type of inoculation. Studies indicate that *Bacillus* sp. BT3 and *Klebsiella* sp. HA9 reduces proline and increases sugars under drought. PGPB improves drought tolerance by increasing sugars and reducing proline. Discrepancies in osmoregulation arise from bacterial variations, communication mechanisms, and the complexity of the metabolic response to stress [20],[24],[56],[57].

Protein concentration decreased in uninoculated wheat plants under severe drought stress, likely due to a reduction in nutrients required for protein synthesis [12]. In contrast, inoculation with PGPB increased protein content by attenuating the effects of stress, promoting nitrogen uptake. These results are consistent with previous studies showing an intensification of proteins under inoculation, highlighting the beneficial role of PGPB in the preservation of cellular processes in the face of environmental constraints [58].

Antioxidant enzymes are proving to be an effective strategy for increasing drought tolerance. Our results demonstrate that water stress increases the production of antioxidant enzymes such as GPX, CAT and SOD. A significant improvement in the activity of antioxidant enzymes is observed following inoculation with strains ME1, Pa, MR6 and D13, particularly under conditions of severe water deficit (25% FC), compared to the non-control inoculated. These results provide evidence for the beneficial effect of inoculation with PGPB to improve drought tolerance by adjusting the activities of antioxidants and detoxification of reactive oxygen species (ROS) in drought conditions. Inoculation of wheat plants with *B. subtilis* resulted in an increase in the activity of antioxidant enzymes (SOD, CAT, and POD) according to [59]. *B*. *amyloliquefaciens* QST713 increases the activity of antioxidant enzymes (SOD, CAT, POD, and APX leading to a decrease in H_2_O_2_ and O_2_−content promoting growth and biomass in alfalfa plants (*Medicago sativa* L.) under conditions of water stress [60]. However, in some cases, a decrease in the activity of antioxidant enzymes in wheat under water stress conditions was observed after inoculation with PGPB [19],[49]. Interactions between microorganisms and plants are diverse and can positively influence plant growth in various ways. Thus, PGPB can improve plant growth through several mechanisms, including the induction of systemic resistance.

The efficiency of root colonization by these strains was visualized using TTC staining and microscopic observation of the roots in which the intensity of the color was proportional to the bacterial colonization. Analysis of the results revealed dense and compact colonization on the surface of wheat roots, both under normal conditions and under water stress. This efficient root colonization capacity is essential for the survival and promotion of plant growth under stressful conditions. These visual observations consolidate the quantitative results obtained, which indicate that the bacterial strains, in particular Pa and D13, are capable of maintaining efficient root colonization, even in the presence of osmotic stress (10% PEG) revealing their osmotolerant potential. The ability of these strains to effectively colonize roots is attributed to their survival and attachment to the rhizoplane, thereby promoting their growth in a drought-prone environment. The complex mechanisms of colonization involve processes such as adhesion, chemical communication and secretion of beneficial compounds. Root exudates act as nutrient reservoirs and chemotactic signals for root-associated bacteria [61]. The results obtained are consistent with those found in other studies [19],[61],[62]. This highlights their potential as bioinoculants to improve crop tolerance to water stress.

The studies confirmed that PGPB can effectively colonize the rhizosphere of plants, thereby improving their stress tolerance. The increased root colonization capacity observed under water stress conditions, notably in strains Pa and D13, is attributed to their ability to produce EPS and the formation of *Bacillus* endospores, adaptations favorable to survival in a stressful environment [23]. *P*. *agglomerans* (Pa) strain is recognized as a beneficial endophytic colonizer, associated with increased biomass production of wheat plants under normal and stressed conditions [27],[37]. In sum, these results highlight the importance of PGPB bacteria establishing close relationships with plants for maximum effectiveness, especially in harsh environments [63].

## 5. Conclusions

The main objective of this research was based on the exploitation of indigenous strains of PGPB present in soils subjected to stress. These multi-tolerant bacteria offered considerable potential as biofertilizers to promote the growth of wheat plants in environments characterized by drought. The results of this study highlighted that the strains, namely ME1, Pa, MR6 and D13, exhibited various PGP characteristics. Furthermore, these strains demonstrated their ability to tolerate different types of stress, thus reinforcing their potential for application in varied environmental ecosystems. When used to inoculate germinating seeds and promote wheat growth under water stress conditions, these strains significantly attenuated the adverse effects of drought, as demonstrated by various parameters related to germination, plant growth, and physiology. The strains also demonstrated their ability to colonize wheat roots both in the presence and absence of stress.

Additional field experiments, particularly in semi-arid and arid soils, are recommended to validate these results and demonstrate their effectiveness in real-world conditions. Future research should focus on developing effective microbial formulations with extended shelf life to promote sustainable agricultural practices in drylands, thereby significantly reducing the reliance on chemical fertilizers and pesticides.

Nevertheless, the commercial viability of PGPB relies largely on the robustness of the strains, the shelf life of the formulations, and their cost-effectiveness. Despite the difficulties encountered, it is essential not to underestimate the hope associated with the positive effects of PGPB in alleviating drought in plants. Thus, it is imperative to focus more efforts on the commercialization of PGPB-based formulations, to make effective solutions available to farmers around the world. This will help improve agricultural practices in arid regions.

## Use of AI tools declaration

The authors declare they have not used Artificial Intelligence (AI) tools in the creation of this article.


