# Supplementary material for: Osmotolerant plant growth promoting bacteria mitigate adverse effects of drought stress on wheat growth
Source: AIMS Microbiol. 2024 Jul 9;10(3):507–41. doi: 10.3934/microbiol.2024025 (PMC11362268; doi:10.3934/microbiol.2024025)
Supplement: Supplementary file 1 [file microbiol-10-03-025-s001.pdf]

AIMS Microbiology

AIMS Microbiology, 10(3): 507–541.
DOI: 10.3934/microbiol.2024025
Received: 02 March 2024
Revised: 07 June 2024
Accepted: 25 June 2024
Published: 09 July 2024

https://www.aimspress.com/journal/microbiology

Research article

Osmotolerant plant growth promoting bacteria mitigate adverse effects of drought stress on wheat growth

Naoual Bouremani[1], Hafsa Cherif-Silini[1,*], Allaoua Silini[1], Nour El Houda Rabhi[2], Ali Chenari Bouket[3] and Lassaad Belbahri[4]

[1] Laboratory of Applied Microbiology, Department of Microbiology, Faculty of Natural and Life Sciences, University Ferhat Abbas of Setif-1, 19000 Setif, Algeria

[2] Department of Natural Sciences and Life, Abdelhafid Boussouf University Center-Mila, 43000 Mila, Algeria

[3] East Azarbaijan Agricultural and Natural Resources Research and Education Centre, Plant Protection Research Department, Agricultural Research, Education and Extension Organization (AREEO), Tabriz 5355179854, Iran

[4] University Institute of Teacher Education (IUFE), University of Geneva, 24 Rue du Général-Dufour, 1211 Geneva, Switzerland

* **Correspondence:** Email: cherifhafsa@univ-setif.dz, cherifhafsa@yahoo.fr; Tel: +213771451451.

Supplementary

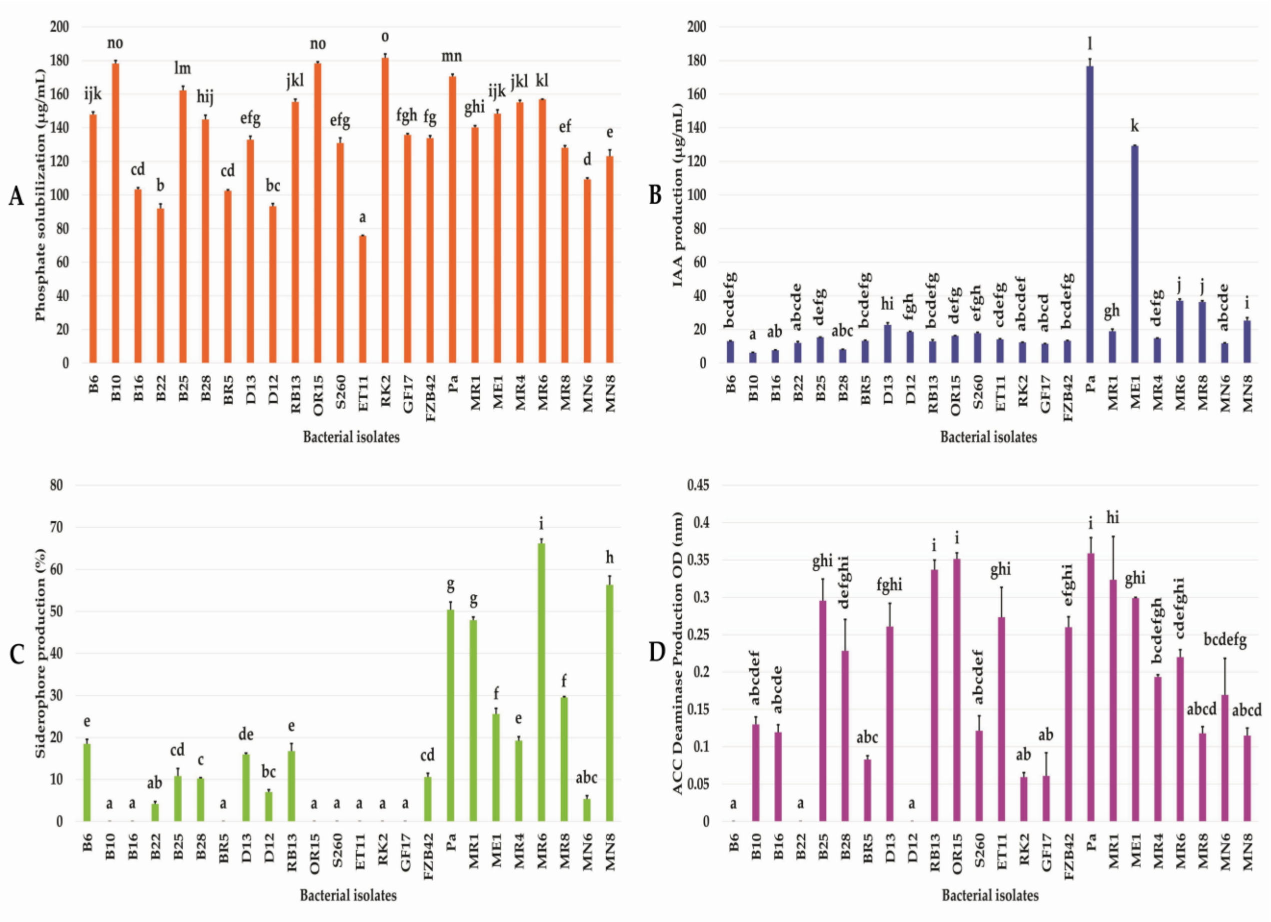

Figure S1. PGP activities of bacterial isolates, (A) Phosphate solubilization (µg/mL), (B) Indole acetic acid (IAA) production (µg/mL), (C) Siderophores production (%) and (D) 1-Aminocyclopropane-1-carboxylate (ACC) deaminase production (DO). Values are means ± standard deviation (SD) of three replicates (n = 3).

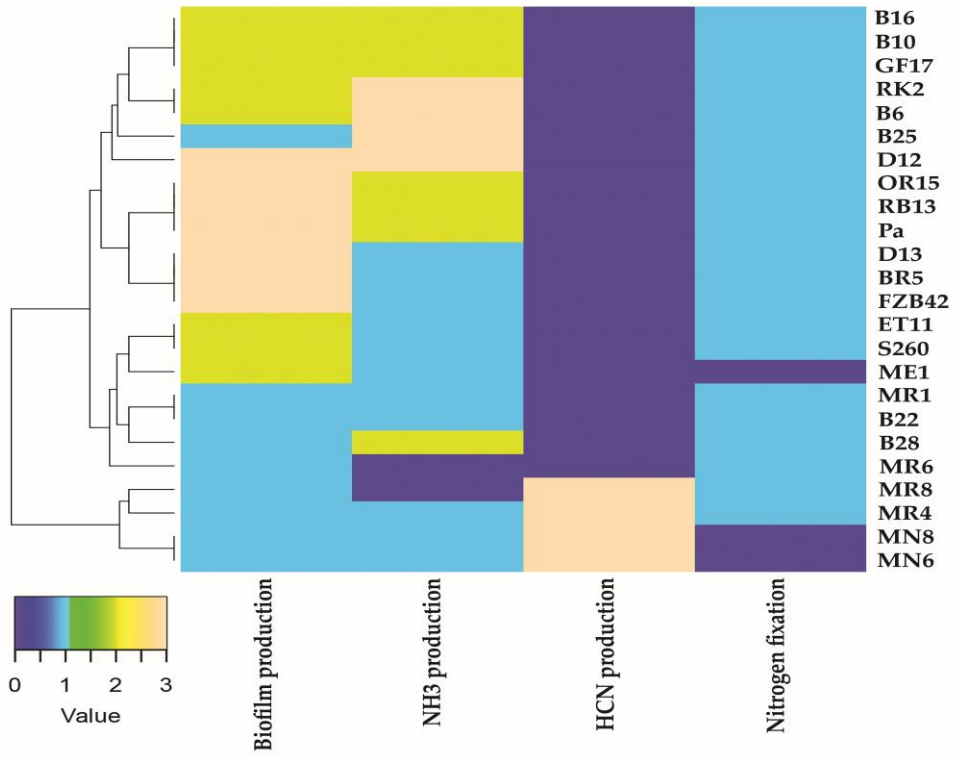

Figure S2. Clusters and heat map of PGP activities (biofilm, NH_3, HCN production and N_2 fixation) of bacterial isolates.

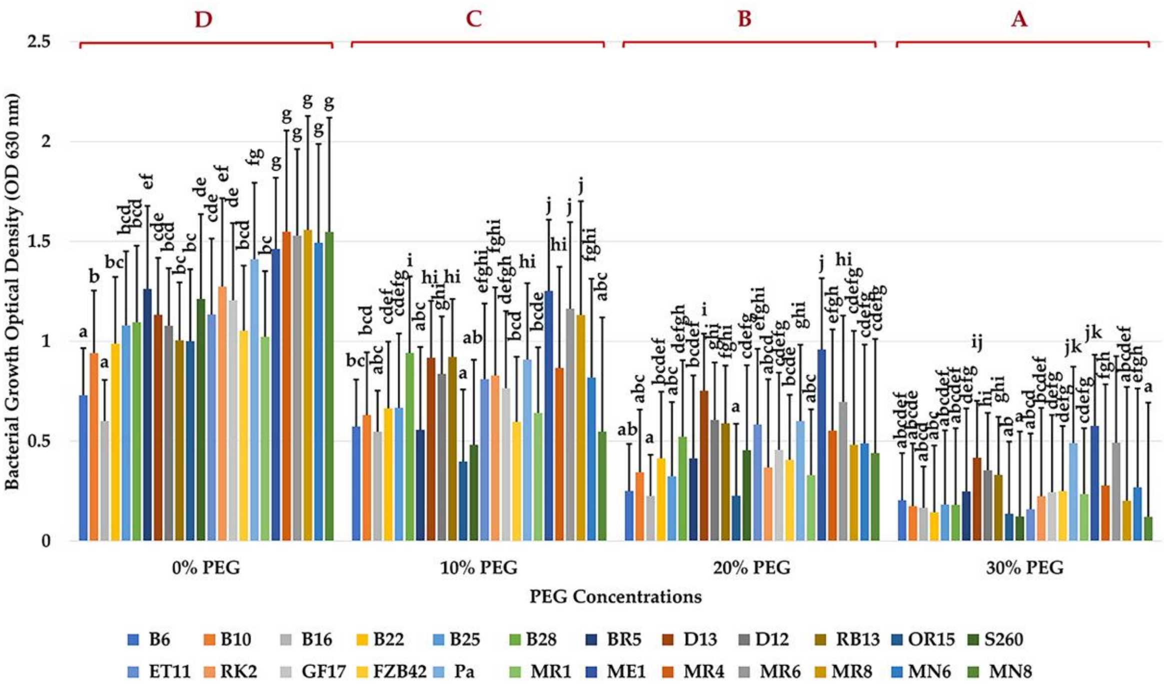

Figure S3. Growth performance of bacterial isolates at different levels of osmotic stress using polyethylene glycol (PEG-6000).

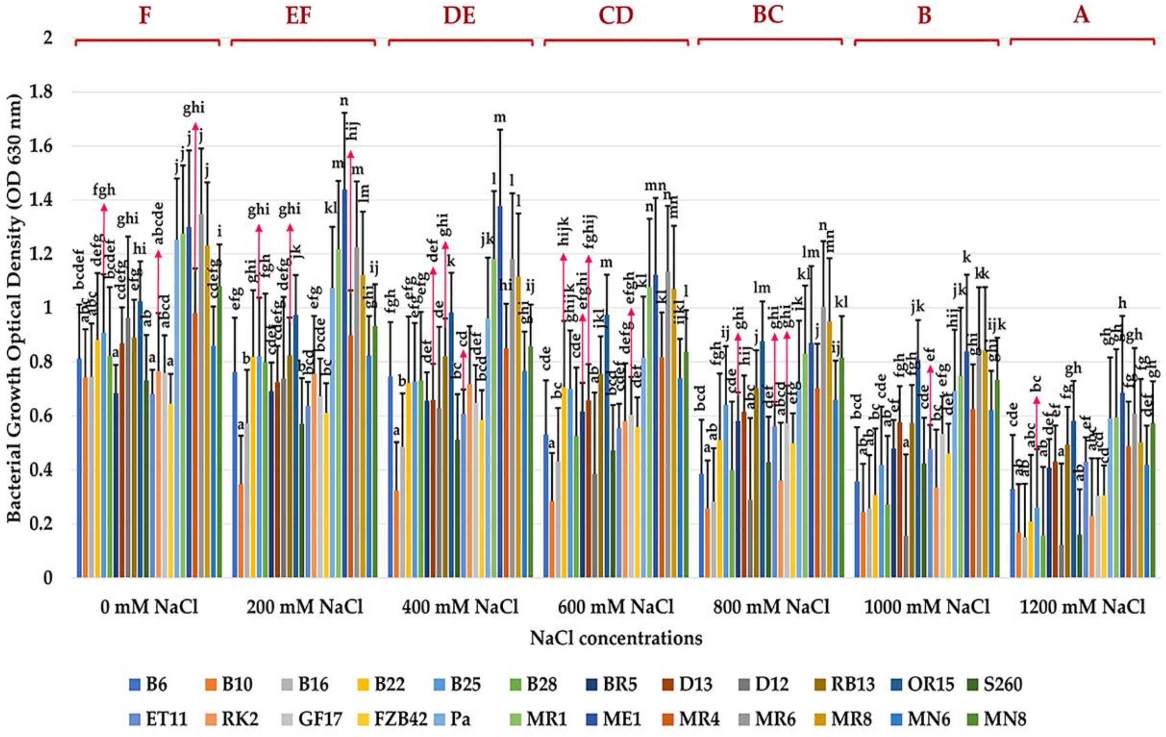

Figure S4. Growth performance of bacterial isolates at different concentrations of NaCl.

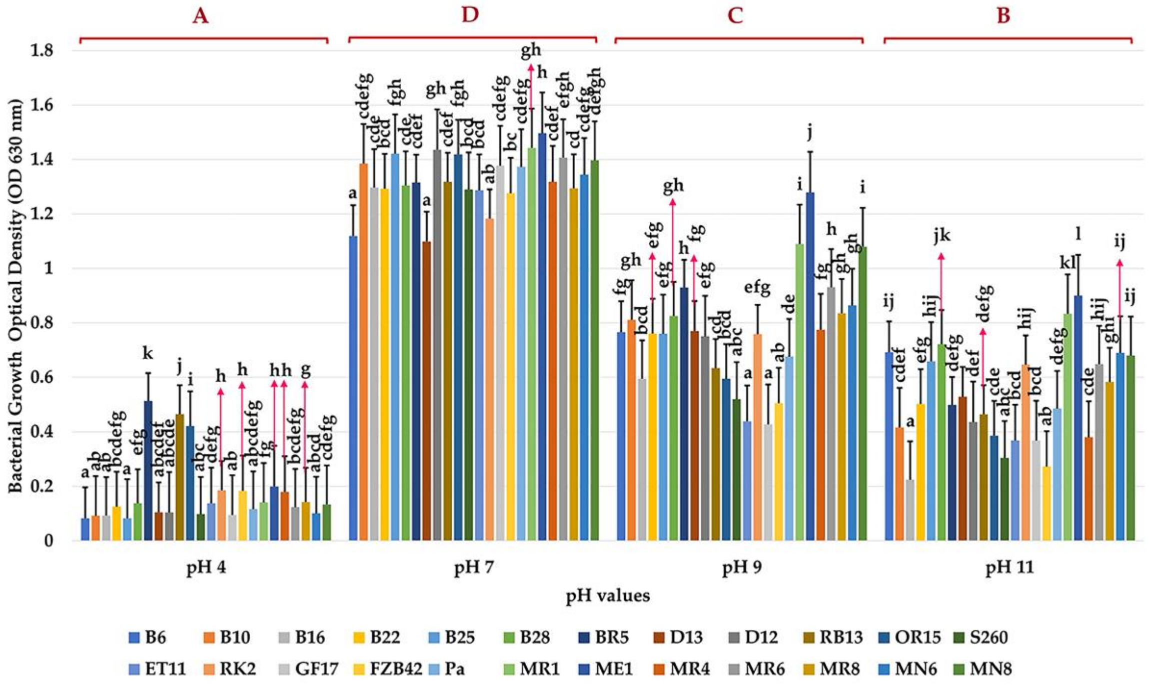

Figure S5. Growth performance of bacterial isolates at different pH values.

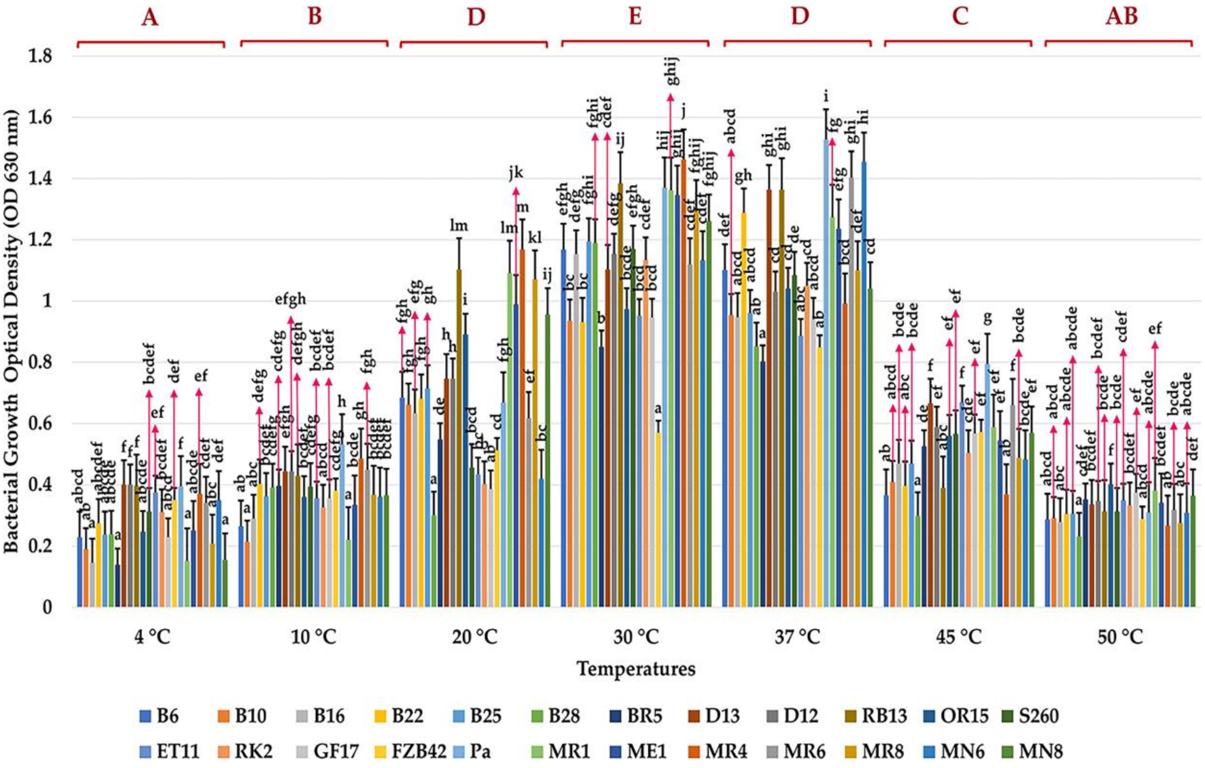

Figure S6. Growth performance of bacterial isolates at different temperatures.

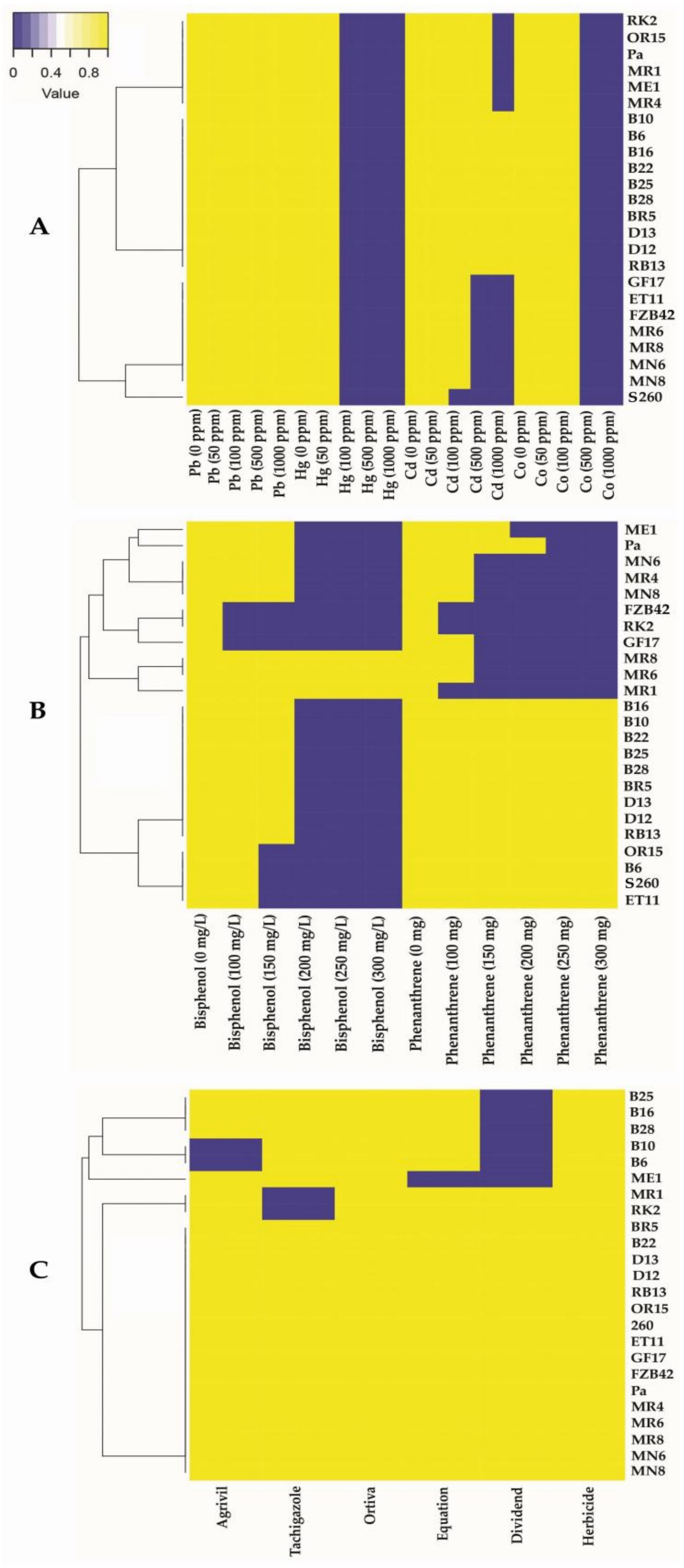

Figure S7. Clusters and heat map of bacterial isolates tolerance to (A) different concentrations of heavy metals (Hg, Pb, Cd, and Co) (B) pollutants (phenanthrene and bisphenol), (C) Herbicides, and fungicides (Agrivil, Tachigazole, Ortiva, Equation and Dividend).

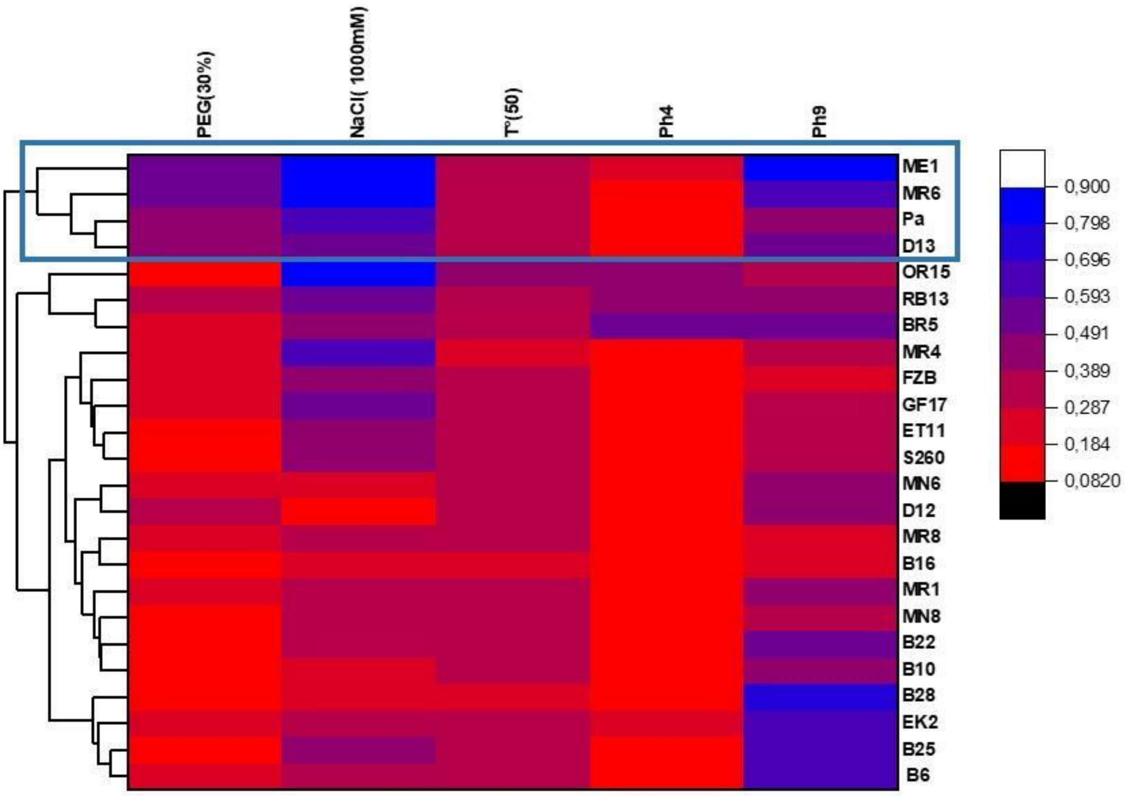

Figure S8. Heat map showing the response of the isolates to different stress tolerance (pH 4, 9, temperature 50 °C, NaCl 1000 mM, and PEG 30%). The bacterial isolates (ME1, MR6, Pa and D13) having the best capacities are highlighted with the same colors.

AIMS Press

© 2024 the Author(s), licensee AIMS Press. This is an open access article distributed under the terms of the Creative Commons Attribution License (https://creativecommons.org/licenses/by/4.0)
